# The lncRNA MIR181A1HG in extracellular vesicles derived from highly metastatic colorectal cancer cells promotes liver metastasis by remodeling the extracellular matrix and recruiting myeloid-derived suppressor cells

**DOI:** 10.1186/s13578-025-01365-2

**Published:** 2025-02-19

**Authors:** Yichao Gu, Yushuai Mi, Yifan Cao, Kuan Yu, Zihao Zhang, Peng Lian, Dawei Li, Jing Qin, Senlin Zhao

**Affiliations:** 1 Department of General Surgery, Zhongshan Hospital, 180 Fenglin Road, Shanghai, 200032 China; 2 Department of Gastrointestinal Surgery, The Second Hospital, Cheeloo College of Medicine, Shandong University, No. 247 Beiyuan Street, Jinan, Shandong 250033 China; 3 Department of Colorectal Surgery, Fudan University Shanghai Cancer Center, 270 Dong’an Road, Shanghai, 200032 China; 4 Department of Oncology, Shanghai Medical College, Fudan University, 270 Dong’an Road, Shanghai, 200032 China

**Keywords:** LncRNA MIR181A1HG, Colorectal liver metastasis, Hepatic stellate cells, Extracellular matrix, Myeloid-derived suppressor cells

## Abstract

**Background:**

Colorectal liver metastasis (CRLM) is the main cause of death in colorectal cancer (CRC) patients worldwide. In the initial stage of metastasis, primary tumors provide the necessary conditions for metastasis by shaping the local microenvironment of the target organ, forming “premetastatic niches” (PMNs), and extracellular vesicles (EVs) play important roles in shaping PMNs. Therefore, investigating the EVs involved in the regulation of PMNs and their mechanism is highly valuable for the further understanding of CRLM.

**Methods:**

Transmission electron microscopy and differential ultracentrifugation were used to verify the existence of exosomes. In vivo and in vitro assays were used to identify the roles of MIR181A1HG in EVs in CRLM. RNA pull-down and dual-luciferase reporter assays were used to clarify the mechanism by which MIR181A1HG in EVs regulated the crosstalk between CRC cells and hepatic stellate cells (HSCs).

**Results:**

We demonstrated that the lncRNA MIR181A1HG was progressively upregulated in tissues, serum EVs from healthy normal controls to CRC and paired liver metastatic groups. Additionally, we verified that HNRNPA2B1 mediated the packaging of MIR181A1HG into CRC cell-derived EVs, which in turn functioned as a ceRNA by sponging miR373-3p to activate HSCs via the TGFβRII/Smad2/3 signaling pathway. Furthermore, activated HSCs could secrete the chemokine CXCL12 to promote CRLM by remodeling the extracellular matrix and recruiting myeloid-derived suppressor cells in the liver, which resulted in liver metastasis.

**Conclusions:**

MIR181A1HG in EVs from highly metastatic CRC cells promoted CRLM by activating HSCs to form PMNs in the liver, which contributes to the further understanding of the mechanism of CRLM and provides potential predictive markers for CRLM.

**Supplementary Information:**

The online version contains supplementary material available at 10.1186/s13578-025-01365-2.

## Background

Colorectal cancer (CRC) accounts for approximately 10% of all annually diagnosed cancers and cancer-related deaths worldwide [1]. Despite increasing advances in treatment, mortality from CRC, especially from metastatic CRC, remains high among cancer-related deaths [2]. The liver is the most frequent site of metastasis for CRC, with approximately 50% of individuals with colorectal liver metastases (CRLMs) presenting with synchronous or metachronous liver metastases [3]. Liver metastasis (LM) is also dependent on the formation of “prometastatic” niches (PMNs) that support the spread of primary tumor cells to the liver [4]. A better understanding of the mechanisms that direct the formation of PMNs is crucial for designing therapeutic strategies and improving the cure rates for CRLM.

On the basis of Paget’s “seed and soil” hypothesis, formation of the PMN requires communication between tumor cells and stromal cells in the tumor microenvironment (TME) to promote tumor progression [5]. In response to tumor invasion of the liver, hepatic stellate cells (HSCs), which are resident liver-specific pericytes, are activated into tumor-associated myofibroblasts that are critical for PMN formation in the liver [6, 7] by altering the quality and quantity of the extracellular matrix (ECM) and producing the characteristic type I collagen-rich “scar” matrix [8]. In addition, extracellular vesicles (EVs) have been identified in the TME, and increasing evidence suggests that they play crucial roles in cancer development, including PMN formation and metastasis [9]. EVs are small membrane vesicles containing functional biomolecules (proteins, lipids, RNA and DNA) that can be horizontally transferred to recipient cells, serving as tumor-derived factors that induce vascular leakiness, inflammation and bone marrow progenitor cell recruitment during PMN formation and LM [10]. We previously demonstrated the significant crosstalk effects of highly metastatic colorectal cancer cells and hepatic stellate cells on CRLM, which were regulated by miR-181a-5p-rich extracellular vesicles released from CRC cells. Noncoding RNAs (ncRNAs), particularly long noncoding RNAs (lncRNAs) and microRNAs (miRNAs), play significant roles in epigenetic regulation [11, 12]. Recently, a complex interplay between these two classes of regulatory ncRNAs has been discovered, in which some lncRNAs can produce miRNAs that repress target mRNAs [13, 14]. For example, the lncRNA H19-derived miR-675 suppresses the translation of insulin growth factor receptor (Igf1r), inhibiting cell proliferation in response to cellular stress or oncogenic signals [15]. miR-17∼92, generated from the lncRNA MIR17HG locus, attenuates TGF-β signaling to stimulate angiogenesis and tumor growth [16]. The lncRNA MIR181A1HG, also known as the main gene of the miR181a/b-1 cluster, can also regulate the expression of precursor miRNAs (premiR181a-1, premiR181b-1) and the corresponding mature miRNAs (miR-181a-3/5p, miR-181b-3/5p). However, whether MIR181A1HG, the main gene of the corresponding mature miRNA181a-5p, contributes to the progression of CRC and liver metastasis is unclear. Accordingly, on the basis of our previous work, we conducted the following series of experiments.

In this study, we discovered that highly metastatic CRC cells released MIR181A1HG-rich EVs that activated HSCs, which ultimately contributed to facilitating CRLM by remodeling the ECM and recruiting myeloid-derived suppressor cells (MDSCs) during liver metastasis. These findings identified a novel specific biomarker for CRLM and a new strategy for predicting the risk of secondary liver cancer caused by CRC.

## Materials and methods

### Patients and tissue specimens

A total of 90 patient-derived specimens were collected and archived under protocols approved by the institutional review boards of Fudan University Shanghai Cancer Center (FUSCC), and their clinicopathological characteristics are summarized in Supplementary Table S1. All patients from the clinical cohorts did not receive any treatment for cancer before surgery and had no other cancers. Human serum samples were collected prior to tumor resection, whereas normal mucosa and paired cancer tissues, as well as metastatic tissues, were collected immediately after surgical resection. Written informed consent was obtained from each patient, and the study protocol conformed to the ethical guidelines of the 1975 Declaration of Helsinki and was approved by the Ethics Committee of our institution (ID: 050432-4-1911D).

### EV isolation and analysis

To remove any cell contamination, the supernatant collected from 3-day cell cultures or plasma samples was first centrifuged at 500 × g for 10 min. Subsequently, the supernatant was centrifuged at 12,000 × g for 20 min to remove any possible apoptotic bodies or large cell debris. The EVs were subsequently enriched by centrifugation at 100,000 × g for 70 min. Finally, they were rinsed in 20 ml of PBS and collected by ultracentrifugation at 100,000 × g for 70 min. The extracellular vesicle cup-shaped morphology and number were evaluated with a Philips CM120 BioTwin transmission electron microscope (FEI Company, USA) and a NanoSight NS300 (Malvern Instruments Ltd., UK), respectively.

Other materials and methods used in this study are described in the supplementary materials.

## Results

### The lncRNA MIR181A1HG is overexpressed in CRLM, which predicts poor prognosis

**Fig. 1:**
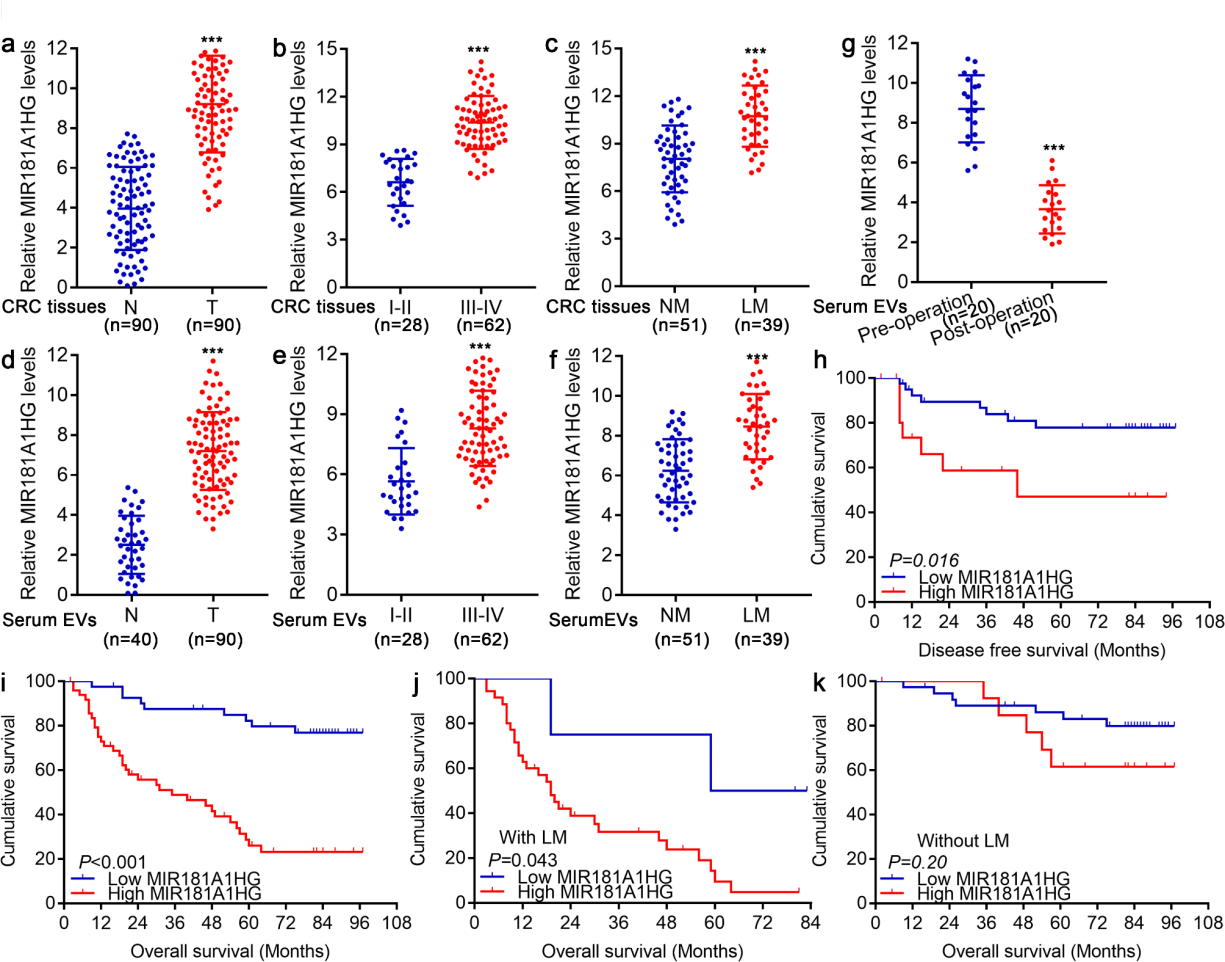
The lncRNA MIR181A1HG is overexpressed in CRLM, predicting poor prognosis. (**a**) The expression levels of MIR181A1HG in 90 CRC tissues and paired adjacent normal colorectal mucosa samples were determined by qPCR. (**b**) The expression levels of MIR181A1HG in CRC tissues from patients at stages I-II (*n* = 28) and III-IV (*n* = 62) were determined by qPCR. (**c**) qPCR analysis of the expression levels of MIR181A1HG in CRC patients without liver metastasis (NM, *n* = 51) and those with liver metastasis (LM, *n* = 39). (**d**) qPCR assays were used to assess MIR181A1HG expression in serum EVs from CRC patients (*n* = 90) and healthy controls (*n* = 40). (**e**) qPCR analysis of the expression levels of MIR181A1HG in serum EVs from CRC patients at stages I-II (*n* = 28) and III-IV (*n* = 62). (**f**) qPCR assays were used to evaluate MIR181A1HG expression in serum EVs from CRC patients without liver metastasis (NM, *n* = 51) and those with liver metastasis (LM, *n* = 39). (**g**) qPCR was used to assess MIR181A1HG expression in the serum EVs of 20 patients with CRLM before radical resection as well as one week after simultaneous resection of CRC primary tumors and LMs. Kaplan–Meier survival curves of CRC patients according to MIR181A1HG expression. CRC patients with a higher MIR181A1HG expression had obvious poorer disease-free survival (**h**) and overall survival (**i**) rates than those with low MIR181A1HG expression. Comparisons of overall survival between the MIR181A1HG-high and MIR181A1HG-low expression samples from CRC patients with (**j**) or without (**k**) LM. (***P < 0.001)

In our preliminary study, we demonstrated that highly metastatic CRC cells released miR-181a-5p-rich extracellular vesicles that could promote liver metastasis by activating hepatic stellate cells and remodeling the tumor microenvironment. The lncRNA MIR181A1HG, also known as the main gene of the miR181a/b-1 cluster, can regulate the expression of precursor miRNAs (premiR181a-1, premiR181b-1) and the corresponding mature miRNAs (miR-181a-3/5p, miR-181b-3/5p). However, the role of MIR181A1HG in CRLM remains unclear. In the present study, we examined the expression levels of MIR181A1HG in 90 pairs of CRC tissues and matched adjacent normal tissues from the FUSCC dataset and found that its expression was apparently higher in CRC tissues than in adjacent normal tissues (Fig. 1a). Moreover, MIR181A1HG expression was significantly higher in stage III-IV CRC patients than in stage I-II CRC patients (Fig. 1b). More importantly, CRC patients with LM presented marked overexpression of MIR181A1HG compared with those without LM (Fig. 1c). We also assessed MIR181A1HG expression in EVs from serum samples from the same CRC cohorts and 40 healthy donors and discovered that MIR181A1HG expression was obviously elevated in EVs derived from CRC patients compared with those from healthy donors (Fig. 1d), and its expression was also obviously greater in EVs derived from CRC patients at stages III-IV than in EVs derived from CRC patients at stages I-II (Fig. 1e). Notably, the expression levels of MIR181A1HG in EVs from CRC patients with LMs were significantly greater than those in EVs from patients without LMs (Fig. 1f). The MIR181A1HG expression of 90 CRC patients was then averaged, and values above the mean value were defined as high expression, whereas those below the mean value were defined as low expression. Associations between MIR181A1HG expression and clinicopathological features were further verified, and the results revealed that the overexpression of MIR181A1HG was significantly correlated with distant metastasis as well as advanced pathological stage (Supplementary Tables S4, 5). More interestingly, we further assessed MIR181A1HG expression in the serum EVs of 20 patients with CRLM before radical resection as well as one week after simultaneous resection of primary CRC tumors and LMs and found that the expression level of MIR181A1HG in the serum EVs of the same patient was significantly decreased one week after surgery (Fig. 1g, Supplementary Table S6). Subsequently, Kaplan–Meier analysis with the log-rank test also revealed that high MIR181A1HG expression was associated with poorer disease-free survival (DFS) and overall survival (OS) in CRC patients (Fig. 1h, i). Notably, high MIR181A1HG expression was associated with a significant reduction in OS only in the liver metastasis group (Fig. 1j, k). In addition, analyses of The Cancer Genome Atlas (TCGA) dataset revealed that MIR181A1HG was also significantly upregulated in CRC tissues and that high MIR181A1HG expression indicated poor recurrence-free survival in CRC patients (Supplementary Fig. S1a-c). Collectively, these results demonstrated that high MIR181A1HG expression plays a significant role in the progression of CRC patients, especially patients with CRLM.

### MIR181A1HG in CRC cell-derived EVs promotes CRLM by inducing liver premetastatic niche formation

**Fig. 2:**
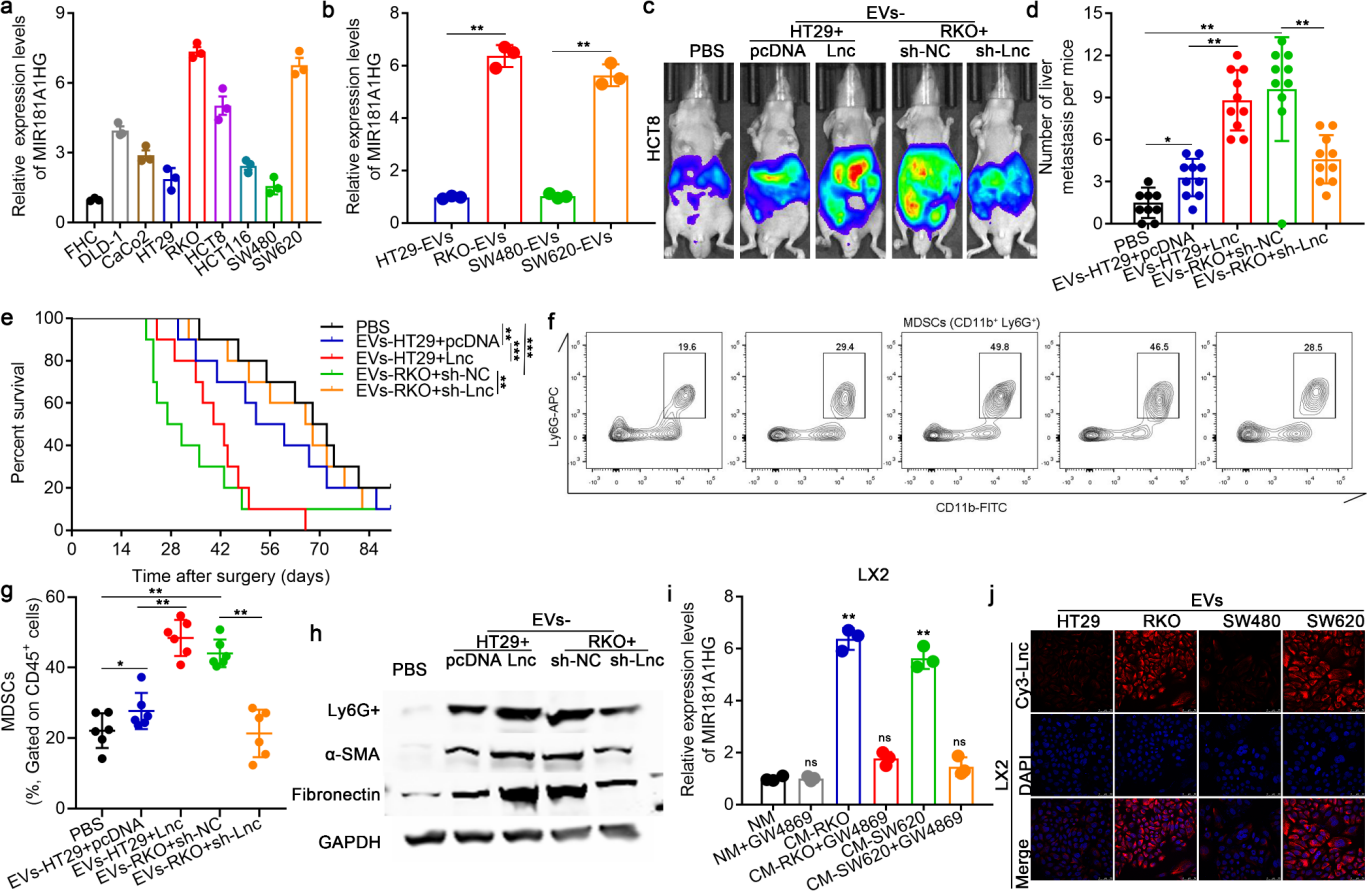
MIR181A1HG in CRC cell-derived EVs promotes CRLM by inducing liver premetastatic niche formation. (**a**) The expression levels of MIR181A1HG in 8 CRC cell lines and 1 normal colorectal mucosa cell line (FHC) were assessed by qPCR. (**b**) qPCR assays were used to evaluate MIR181A1HG expression in EVs derived from 4 CRC cell lines, HT29 and SW480, which possess relatively weak metastatic potential, and RKO and SW620, which possess relatively high metastatic potential. (**c**) HT29 and RKO CRC cells were transfected with MIR181A1HG overexpression or knockdown constructs, respectively; luciferase-labeled HCT8 CRC cells were then injected into the spleens of nude mice to construct mouse models of CRLM, followed by injection of EVs into the tail vein. The impact of EVs derived from CRC cells on CRLM in vivo was determined via spleen injection to construct mouse models of CRLM. **c.** Representative live images of LMs in nude mice. (**d**) Number of metastatic colonies in the livers of the nude mice from different groups determined by live imaging and HE staining. (**e**) Overall survival of each group of mice (*n* = 10 for each group). (**f**) Representative flow cytometry analysis of MDSCs. (**g**) Quantification of MDSCs in each group of mice. (**h**) The expression levels of Ly6G+, α-SMA and fibronectin in the LM tissues of each group were assessed by western blotting. (**i**) qPCR assays were used to determine the expression levels of MIR181A1HG in LX2 cells, which were incubated with conditioned medium (CM) from RKO and SW620 cells with or without EV depletion via an inhibitor of EV secretion (GW4869). (**j**) Representative immunofluorescence staining of LX2 cells that were cocultured with EVs derived from HT29, RKO, SW480, and SW620 cells pretransfected with Cy3-tagged MIR181A1HG (red). (^ns^*P*>0.05, *P < 0.05, **P < 0.01, and ***P < 0.001)

To further explore the role of MIR181A1HG in the progression of CRLM, we first measured MIR181A1HG expression in the conditioned medium (CM) of seven CRC cell lines and found that the expression levels of MIR181A1HG in the CM of HT29 and SW480 cells were relatively low, whereas the expression levels of MIR181A1HG in the CM of RKO and SW620 cells were relatively high (Fig. 2a). Next, we evaluated MIR181A1HG expression in the nucleus, cytoplasm, and CM of four CRC cell lines, HT29, SW480, RKO, and SW620. We observed that the CM of RKO and SW620 cells presented the highest expression level of MIR181A1HG, and the nuclei of these cells presented the lowest MIR181A1HG expression (Supplementary Fig. S2a-d). Notably, the levels of MIR181A1HG in the CM of CRC cells remained unchanged upon RNase A treatment but significantly decreased following treatment with RNase A plus Triton X-100 (Supplementary Fig. S2e), which indicated that extracellular MIR181A1HG was primarily encapsulated within a membrane rather than directly released. Interestingly, MIR181A1HG levels were almost equal between EVs and whole CRC cell CM (Supplementary Fig. S2f). Furthermore, EVs derived from these four CRC cell lines were isolated and purified from the conditioned medium (CM). Electron microscopy and nanoparticle tracking analysis demonstrated that more EVs were secreted from CRC cells with a relatively high expression of MIR181A1HG (Supplementary Fig. S3). Moreover, the expression levels of MIR181A1HG in EVs from highly metastatic CRC cells were significantly greater than those in EVs from weakly metastatic CRC cells (Fig. 2b). These data indicated that MIR181A1HG was encapsulated mainly in EVs derived from CRC cells, especially highly metastatic CRC cells.

To investigate the effects of MIR181A1HG in CRC cell-derived EVs on cancer biological processes in CRLM, the CRC cell line HT29/RKO was transfected with MIR181A1HG overexpression or knockdown plasmids, and then EVs were extracted and cocultured with another CRC cell line, HCT8. Transwell assays were performed to assess the effects of MIR181A1HG in CRC cell-derived EVs on the migration and invasion of CRC cells. However, overexpression or knockdown of MIR181A1HG did not significantly attenuate or strengthen CRC cell migration and invasion, respectively, in vitro (Supplementary Fig. S4a, b). We subsequently injected HCT8 cells into the spleens of BALB/c nude mice to establish a mouse model of CRLM, and the above EVs were subsequently injected into the tail vein. The results revealed more liver metastases and poorer OS following the injection of EVs derived from highly metastatic CRC cells than from the injection of EVs derived from weakly metastatic CRC cells, while the overexpression or knockdown of MIR181A1HG in CRC cell-derived EVs could partially strengthen or attenuate the promoting effects of CRC cell-derived EVs on CRLM (Fig. 2c-e, Supplementary Fig. S4c).

To identify the downstream effectors of MIR181A1HG in CRC cell-derived EVs that are involved in CRLM, Kyoto Encyclopedia of Genes and Genomes (KEGG) analyses were performed on the genes differentially expressed between high and low MIR181A1HG-expressing CRC tumors from TCGA data. We found that these differentially expressed genes were apparently associated with myeloid-derived suppressor cell (MDSC) accumulation, extracellular matrix (ECM) deposition and the TGFβ signaling pathway (Supplementary Fig. S4d). As MDSC accumulation and ECM deposition serve as key components of PMN formation in liver metastasis, we further assessed myeloid infiltration and the expression of biomarkers of MDSCs (CD11b + Ly6G+) and of the ECM (α-SMA and fibronectin) in the aforementioned LM tissues by flow cytometry and western blotting, respectively. Compared with those in the control groups, EVs overexpressing MIR181A1HG presented increased accumulation of MDSCs (Fig. 2f, g), accompanied by increased deposition and expression of Ly6G+, α-SMA and fibronectin in LMs (Fig. 2h). In BALB/c nude mice, the thymus is dysplastic, and immune T cells are absent. To further determine whether immune T cells are involved in MIR181A1HG-mediated regulation of PMN construction in CRLM, we also established a mouse model of CRLM in C57BL/6 mice. The results confirmed that MDSCs rather than T cells were involved in the construction of PMNs during the process of CRLM facilitation by MIR181A1HG in CRC cell-derived EVs (Supplementary Fig. S5).

In a previous study, we demonstrated that CRC cell-derived EVs could activate hepatic stellate cells (HSCs) and remodel PMNs during CRLM [17]. HSCs also serve as key players in remodeling the ECM and the formation of liver PMNs; thus, we chose LX2 cells as a normal HSC line to verify whether MIR181A1HG in CRC cell-derived EVs could be transferred to and activate HSCs, promoting CRLM. We found that the expression levels of MIR181A1HG in LX2 cells incubated with CM from highly metastatic CRC cells were notably greater than those in LX2 cells incubated with CM depleted of EVs with GW4869, an inhibitor of EV secretion (Fig. 2i). Furthermore, following transient transfection with Cy3 (red)-tagged MIR181A1HG, CRC cell-derived EVs were cocultured with LX2 cells, and fluorescently labeled MIR181A1HG was detected in LX2 cells (Fig. 2j), suggesting that MIR181A1HG was transferred from CRC cells to HSCs via EVs, with a greater quantity of MIR181A1HG transferred from highly metastatic CRC cells.

### MIR181A1HG transfer into EVs is regulated by HNRNPA2B

**Fig. 3:**
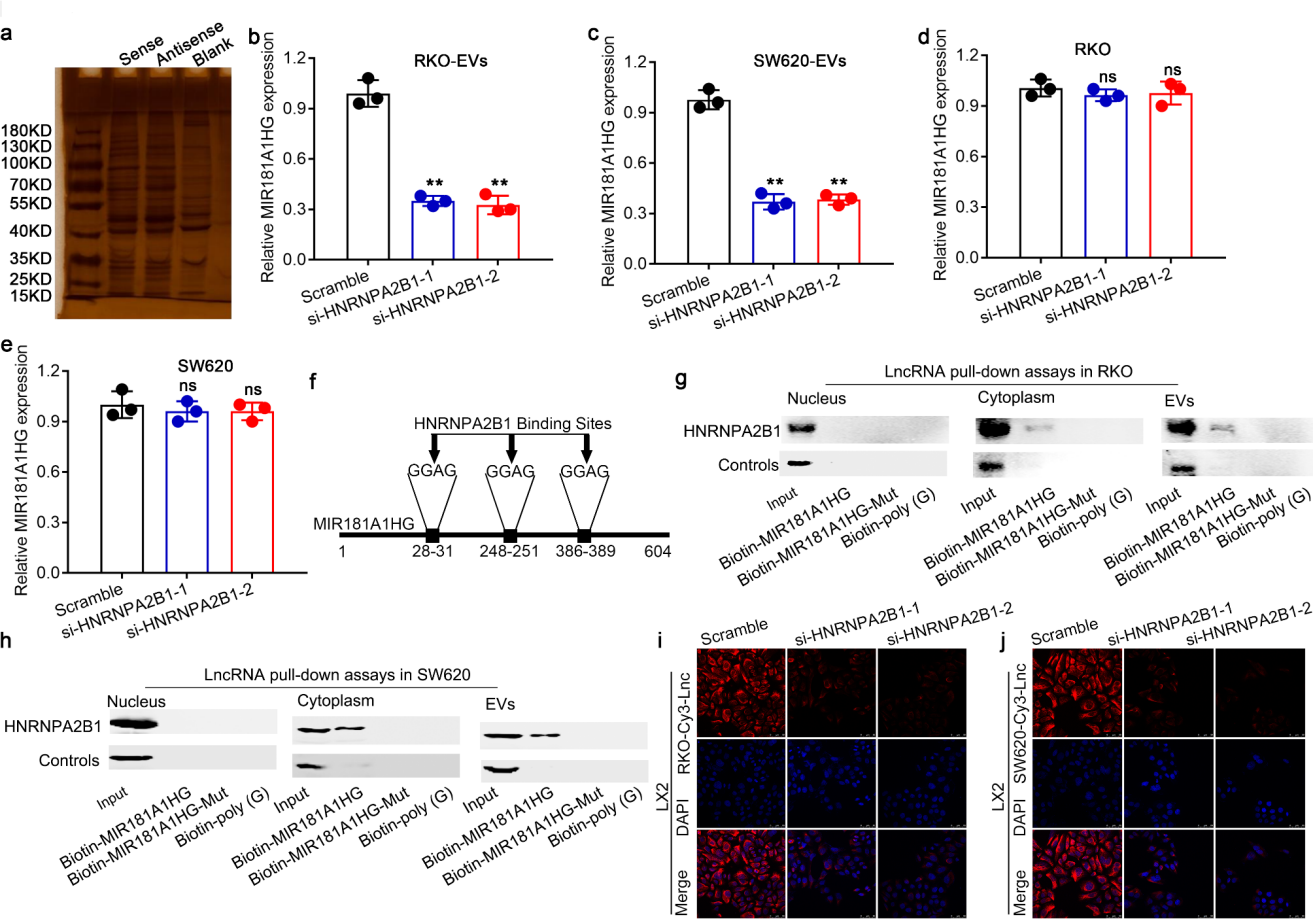
HNRNPA2B mediates the transfer of MIR181A1HG into EVs. (**a**) After biotin-labeled MIR181A1HG, protein samples were prepared by rapid silver staining with pull-down combined protein bands for mass spectrometry analysis and screening of potential RNA-binding proteins responsible for transportation of MIR181A1HG into EVs. (**b**) qPCR assays were used to assess the expression levels of MIR181A1HG in RKO- and SW620-derived EVs **(b**,** c**) and RKO and SW620 cells **(d**,** e**) upon HNRNPA2B1 knockdown. **f.** Schematic diagram of the three specific binding sites of HNRNPA2B1 on MIR181A1HG. WB analysis was used to examine the association between biotinylated wild-type MIR181A1HG/mutant MIR181A1HG and HNRNPA2B1 expression in samples derived by lncRNA pull-down performed with nuclear, cytoplasmic or EV lysates from RKO (**g**) and SW620 (**h**) CRC cells; biotinylated poly(G) was used as the negative control. Representative immunofluorescence images of LX2 cells cocultured with CM from RKO (**i**) or SW620 (**j**) CRC cells transfected with Cy3-MIR181A1HG (red) or si-HNRNPA2B1 are shown. (^ns^*P*>0.05, **P < 0.01)

As EV-mediated transfer of RNAs requires a specific and selective RNA-binding protein for transportation [17], to assess the specific interaction between the MIR181A1HG sequence and the specific RNA-binding proteins regulating MIR181A1HG packaging into EVs, we used RNA pull-down combined with mass spectrometry analysis and found that HNRNPA2B1, IGF2BP2, and HNRNPR could bind to MIR181A1HG in CRC cells (Fig. 3a). We observed that only CRC cells with HNRNPA2B1 expression knocked down presented clearly decreased expression levels of MIR181A1HG in EVs rather than total MIR181A1HG (Fig. 3b-e; Supplementary Fig. S6a-e), whereas the expression levels of HNRNPA2B1 in the four CRC cell lines HT29, RKO, SW480, and SW620 did not significantly differ (Supplementary Fig. S6f). HNRNPA2B1 is essentially an RNA-binding protein that plays an important role in RNA transport and posttranscriptional modification. Furthermore, miRNA pull-down assays revealed that MIR181A1HG and HNRNPA2B1 bound in the cytoplasm and EVs but not to the nucleus, and this binding could be abrogated by mutating the binding sequence (GGAG) in MIR181A1HG (Fig. 3f-h). Subsequently, cell immunofluorescence assays revealed that the process by which MIR181A1HG was transferred from highly metastatic CRC cells to HSCs via EVs could be inhibited by the knockdown of HNRNPA2B1 expression in RKO and SW620 cells (Fig. 3i, j). Therefore, we demonstrated that HNRNPA2B1 could mediate MIR181A1HG packaging into EVs in CRC cells and then transfer EVs containing MIR181A1HG to HSCs by binding to its GGAG sequence.

### MIR181A1HG in CRC cell-derived EVs functions as a ceRNA by sponging miR373-3p to activate LX2 via the TGFβRII/Smad2/3 pathway

**Fig. 4:**
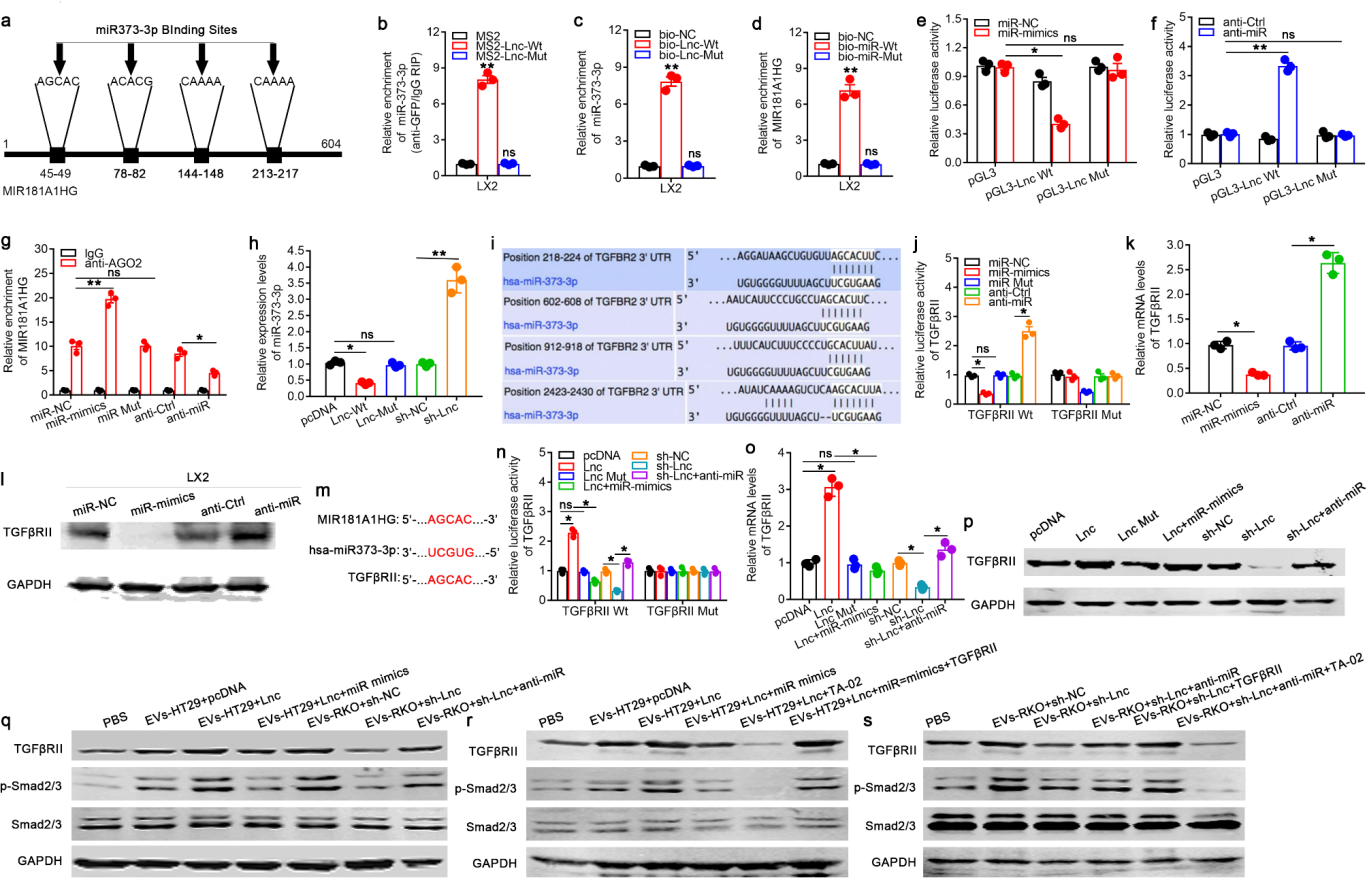
MIR181A1HG in CRC cell-derived EVs functions as a ceRNA by sponging miR373-3p to activate HSCs via the TGFβRII/Smad2/3 pathway. (**a** ) Sequences of the predicted binding sites between MIR181A1HG and miR373-3p. (**b**) MS2-RIP assays followed by qPCR assays were used to measure miR373-3p endogenously associated with MIR181A1HG. LX2 cell lysates were incubated with biotin-labeled MIR181A1HG and miR373-3p, and qPCR analyses of the expression of miR373-3p (**c**) and MIR181A1HG (**d**) in the products of pull-down by biotin were performed. Luciferase reporter gene assays were performed to determine the effects of the miR373-3p mimic (**e**) and anti-miR373-3p (**f**) constructs on the luciferase activity of the wild-type and mutant MIR181A1HG in LX2 cells. **g.** AGO2-RIP assays followed by qPCR assays were used to evaluate the expression levels of MIR181A1HG in LX2 cells transfected with miR373-3p overexpression or knockdown constructs. **h.** qPCR analysis of the expression levels of miR373-3p in LX2 cells transfected with wild-type or mutant MIR181A1HG overexpression or knockdown constructs. **i.** Sequences of the predicted binding sites between miR373-3p and the 3′UTR of wild-type (Wt)/mutant (Mut) TGFβRII. **j.** Luciferase reporter gene assays were performed to determine the effects of the wild-type/mutant-type miR373-3p mimic and anti-miR373-3p on the luciferase activity of wild-type and mutant TGFβRII in LX2 cells. qPCR (**k**) and western blot (**l**) analyses of the expression levels of TGFβRII in LX2 cells transfected with the miR373-3p overexpression or knockdown construct. **m.** MIR181A1HG and TGFβRII share the same miRNA binding site. LX2 cells were first transfected with wild-type and mutant MIR181A1HG overexpression and knockdown constructs and with miR373-3p mimics and anti-miR373-3p constructs. The luciferase activities of wild-type and mutant TGFβRII and the expression levels of TGFβRII were subsequently determined by luciferase reporter gene assays (**n**), qPCR (**o**), and western blot assays (**p**). LX2 cells were first cocultured with EVs derived from HT29 and RKO cells that had been pretransfected with MIR181A1HG-overexpressing or MIR181A1HG-knockdown constructs. Then, LX2 cells were transfected with miR373-3p mimics or anti-miR373-3p. **q.** Western blot analysis of the expression levels of TGFβRII, Smad2/3 and phosphorylated Smad2/3 in the above LX2 cells. **r. s.** Exogenous TGFβRII or TA-02, a potent and selective inhibitor of TGFβRII, was added to the above LX2 cells, and the expression levels of TGFβRII, Smad2/3 and phosphorylated Smad2/3 were subsequently examined by western blot assays. (^ns^*P*>0.05, *P < 0.05, **P < 0.01)

To further explore the mechanisms by which MIR181A1HG in CRC cell-derived EVs is involved in activating HSCs during CRLM, GO/KEGG enrichment analysis of TCGA data revealed that the TGFβ signaling pathway might be involved in the development of CRC, which might be associated with TGFβRII. LncRNAs have been identified as critical regulators of tumor metastasis, and accumulating evidence has demonstrated that lncRNAs regulate miRNA and mRNA expression through competitively sponging miRNAs, and the role of TGF-β/Smads in tumor metastasis has been widely reported [18]. Through TargetScan database analysis and NCBI sequence query base sequence alignment, we identified four potential miR373-3p binding sites on MIR181A1HG, and these sites also exist in the 3’UTR of TGFβRII (Fig. 4a). In view of the above findings, we hypothesized that MIR181A1HG in CRC cell-derived EVs may compete to bind miR373-3p through ceRNA, thereby weakening the latter’s degradation of the 3’UTR of TGFβRII, leading to the activation of TGF-β/Smads, ultimately activating HSCs. We first mutated the binding sites of MIR181A1HG and miR373-3p to construct the MIR181A1HG mut and miR373-3p mut plasmids. Additionally, constructs containing MIR181A1HG (wild-type or mutant) transcripts combined with the MS2 binding site were generated and cotransfected into LX2 cells with a construct containing MS2-binding protein (MS2bp, which binds to the MS2 binding site) and GFP. An anti-GFP RNA immunoprecipitation (RIP) assay was performed, and the results revealed that miR373-3p was enriched only in wild-type MIR181A1HG, whereas the enrichment caused by mut MIR181A1HG was not significant compared with that in the MS2 control (Fig. 4b). These results revealed that miR373-3p specifically bound to MIR181A1HG. To further verify the correlation between miR373-3p and MIR181A1HG, an RNA pull-down assay revealed that miR373-3p could be pulled down by biotin-labeled wild-type MIR181A1HG. Additionally, MIR181A1HG could also be pulled down by biotin-labeled wild-type miR373-3p, whereas miR373-3p was not able to pull down the mutated MIR181A1HG (Fig. 4c, d). These data validated the binding between miR373-3p and MIR181A1HG in LX2 cells. A luciferase reporter construct containing MIR181A1HG (wild-type or mutated miR373-3p binding site) was subsequently generated and cotransfected into LX2 cells with miR373-3p mimics or anti-miR373-3p as well as the corresponding control groups. Moreover, luciferase assays revealed that the overexpression or knockdown of miR373-3p inhibited or enhanced the luciferase activity of wild-type MIR181A1HG, respectively, but did not affect the mutated MIR181A1HG (Fig. 4e, f). AGO2 is essential in miRNA-induced posttranscriptional repression or degradation of RNA to form an RNA-induced silencing complex (RISC) combined with miRNA targets, [14] which was confirmed with an anti-AGO2 RIP assay. Endogenous MIR181A1HG enrichment decreased or increased after the knockdown or overexpression of miR373-3p, respectively, but was not altered by the transfection of mut-miR373-3p (Fig. 4g). These results indicated that MIR181A1HG was recruited to AGO2-related miR373-3p-induced silencing complexes, suggesting that MIR181A1HG interacted with miR373-3p. To further elucidate the correlation between MIR181A1HG and miR373-3p, LX2 cells were transfected with MIR181A1HG overexpression plasmids or shMIR181A1HG. qPCR revealed that miR373-3p expression was reduced or upregulated in MIR181A1HG-overexpressing or MIR181A1HG-silenced LX2 cells, respectively, but was not changed in MIR181A1HG-mut-transfected LX2 cells (Fig. 4h). Together, these data indicated that MIR181A1HG in CRC-derived EVs sponged miR373-3p and might function as a ceRNA in HSCs.

We subsequently constructed luciferase reporter constructs containing the wild-type or mutant TGFβRII 3′UTR as well as mut-miR373-3p with the bases paired with the mut-TGFβRII 3′UTR (Fig. 4i). The luciferase activity of the wild-type but not mutant TGFβRII could be reduced or enhanced by miR373-3p mimics or anti-miR373-3p (Fig. 4j). Furthermore, both the mRNA and protein levels of TGFβRII were downregulated or upregulated after LX2 cells were treated with miR373-3p mimics or anti-miR373-3p (Fig. 4k, l). These results confirmed that TGFβRII was a target of miR373-3p in HSCs.

In the above experiments, we demonstrated that MIR181A1HG might function as a ceRNA by sponging miR373-3p. Interestingly, MIR181A1HG and TGFβRII share the same miR373-3p binding site (Fig. 4m). We also found that miR373-3p regulated TGFβRII expression through binding to the 3′UTR. Consequently, we hypothesized whether MIR181A1HG in CRC cell derived-EVs regulated TGFβRII expression by sponging miR373-3p and promoted the activation of HSCs. To verify this hypothesis, a luciferase assay was performed in LX2 cells cotransfected with the TGFβRII 3′UTR luciferase construct and MIR181A1HG or shMIR181A1HG. The results showed that wild-type MIR181A1HG, but not the mutant MIR181A1HG, increased the luciferase activity of wild-type TGFβRII and that this effect could be attenuated by miR373-3p mimics (Fig. 4n). Moreover, the luciferase activity of mut TGFβRII could not be regulated by MIR181A1HG (Fig. 4n). Conversely, depletion of MIR181A1HG decreased the luciferase activity of wild-type TGFβRII but not mutant TGFβRII, and this decrease was reversed by miR373-3p inhibitors (Fig. 4n). To further understand the regulatory effects of MIR181A1HG in CRC cell-derived EVs on endogenous TGFβRII in LX2 cells, we conducted qPCR and western blot assays to evaluate the effects of the MIR181A1HG in CRC cell-derived EVs on the mRNA and protein levels of TGFβRII. The ectopic expression of wild-type MIR181A1HG, but not the mutant MIR181A1HG, upregulated the mRNA level of TGFβRII, which was attenuated by transfection with miR373-3p mimics (Fig. 4o). In contrast, knockdown of MIR181A1HG downregulated TGFβRII mRNA expression, and anti-miR373-3p partially reversed this downregulation effect (Fig. 4o). In addition, the protein levels of TGFβRII were similar to those of TGFβRII mRNA (Fig. 4p). These data demonstrated that the MIR181A1HG in CRC cell-derived EVs regulated the expression of TGFβRII through a competitive interaction with miR373-3p and the inhibition of miR373-3p activity in HSCs.

Finally, we explored whether MIR181A1HG in CRC cell-derived EVs activated HSCs via the TGF-β/Smads signaling pathway. LX2 cells were first cocultured with EVs derived from HT29/RKO cells, which had been pretransfected with MIR181A1HG overexpression or knockdown constructs. Then, LX2 cells were transfected with miR373-3p mimics or anti-miR373-3p, and the levels of total Smad2/3 and phosphorylated Smad2/3 were measured. The results revealed that CRC cell-derived EVs with MIR181A1HG overexpression or silencing apparently promoted or inhibited the phosphorylation of Smad2/3 in LX2 cells, respectively, whereas the overexpression or knockdown of miR373-3p partially neutralized these effects, respectively (Fig. 4q). Exogenous TGFβRII or TA-02, a potent and selective inhibitor of TGFβRII, was subsequently added to these LX2 cells, and the results further verified that MIR181A1HG in CRC cell-derived EVs activated HSCs via the TGF-β/Smad2/3 signaling pathway (Fig. 4r, s).

### HSCs activated by MIR181A1HG in CRC cell-derived EVs promote CRLM by remodeling the TME

**Fig. 5:**
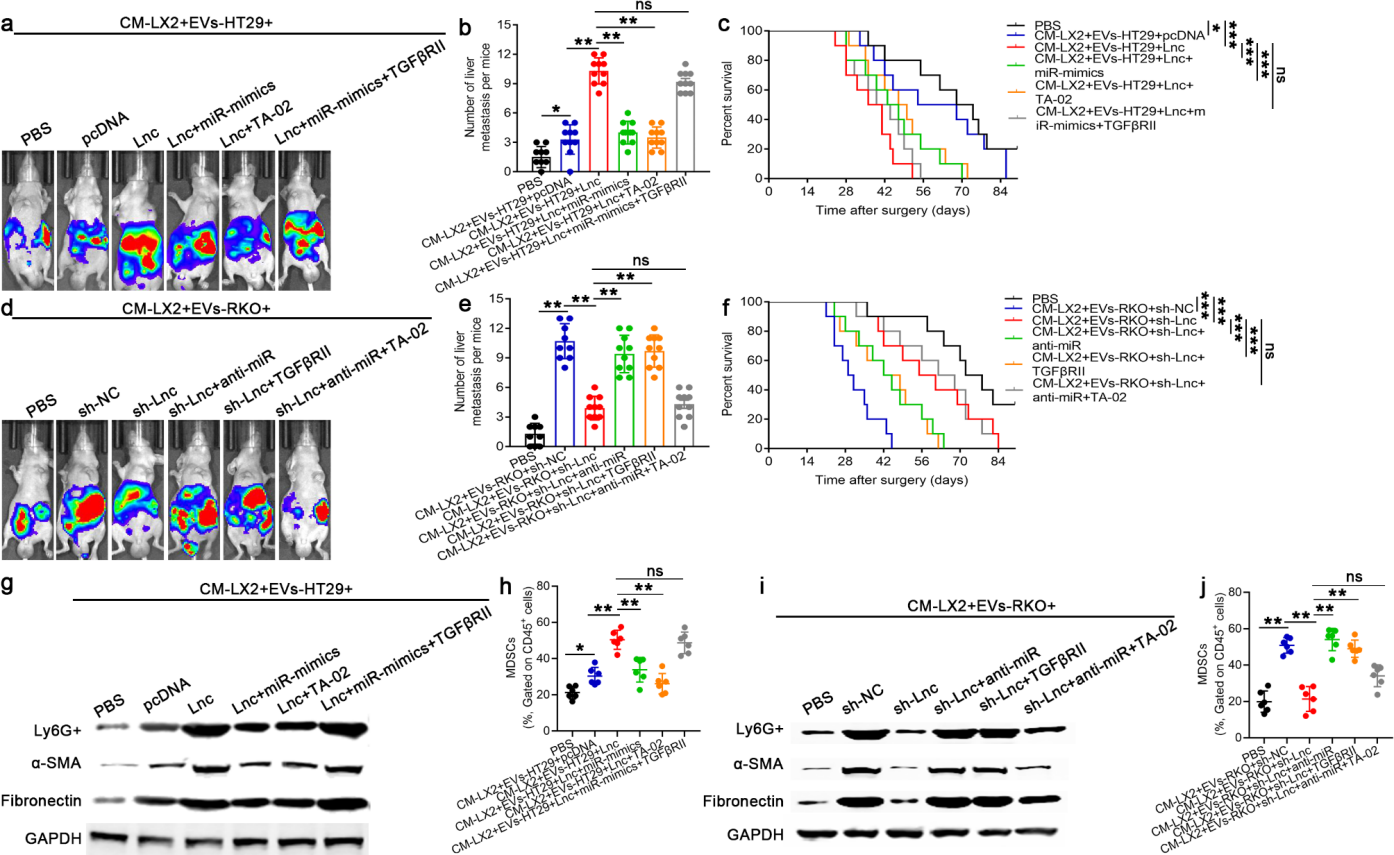
HSCs activated by MIR181A1HG in CRC cell-derived EVs promote CRLM by inducing premetastatic niche formation through recruiting MDSCs and remodeling the ECM in the liver. LX2 cells were first activated by coculturing with EVs derived from HT29 and RKO cells, which had been pretransfected with MIR181A1HG-overexpressing or MIR181A1HG-knockdown constructs. Then, LX2 cells were transfected with miR373-3p mimics or anti-miR373-3p and treated with exogenous TGFβRII or TA-02. Then, luciferase-labeled HCT8 CRC cells were injected into the spleens of nude mice to establish a mouse model of CRLM, and conditioned medium (CM) from activated LX2 cells was injected into the tail vein. The impact of activated HSCs on CRLM in vivo was determined via spleen injection to construct mouse models of CRLM. **a**,** d.** Representative live images of liver metastases in nude mice. **b**,** e.** Number of metastatic colonies in the livers of the nude mice from different groups determined by live imaging and HE staining. **c**,** f.** Overall survival of each group of mice (*n* = 10 for each group). **g**,** i.** The expression levels of Ly6G+, α-SMA and fibronectin in the LM tissues of each group were assessed by western blotting. **h**,** j.** Quantification of MDSCs in each group of mice. (^ns^*P*>0.05, *P < 0.05, **P < 0.01, ***P < 0.001)

To explore whether HSCs activated by MIR181A1HG in CRC cell-derived EVs could promote CRLM, LX2 cells were first cocultured with EVs derived from HT29/RKO cells that had been pretransfected with MIR181A1HG overexpression or knockdown constructs. Then, LX2 cells were transfected with miR373-3p mimics or anti-miR373-3p and treated with exogenous TGFβRII or TA-02, respectively. We injected HCT8 cells into the spleens of nude mice to establish a mouse model of CRLM, and conditioned medium (CM) was injected into the tail vein. More liver metastases and poorer OS were detected following the injection of CM with MIR181A1HG overexpression, whereas miR373-3p overexpression or knockdown, TA-02 or exogenous TGFβRII in LX2 cells partially attenuated or strengthened this process (Fig. 5a-f, Supplementary Fig. S7a, b). We further demonstrated that the ability of MIR181A1HG in CRC cell-derived EVs to promote MDSC accumulation and ECM deposition could be partially attenuated or strengthen by miR373-3p overexpression or knockdown, TA-02 or exogenous TGFβRII in LX2 cells (Fig. 5g-j). These findings indicated that the MIR181A1HG in CRC cell-derived EVs could activate HSCs via the TGF-β/Smad2/3 signaling pathway and that these activated HSCs (α-HSCs) promoted CRLM by recruiting MDSCs and remodeling the ECM in the liver.

### HSCs activated by MIR181A1HG in CRC cell-derived EVs promote CRLM by secreting CXCL12 to recruit MDSCs and remodel the ECM in the liver

**Fig. 6:**
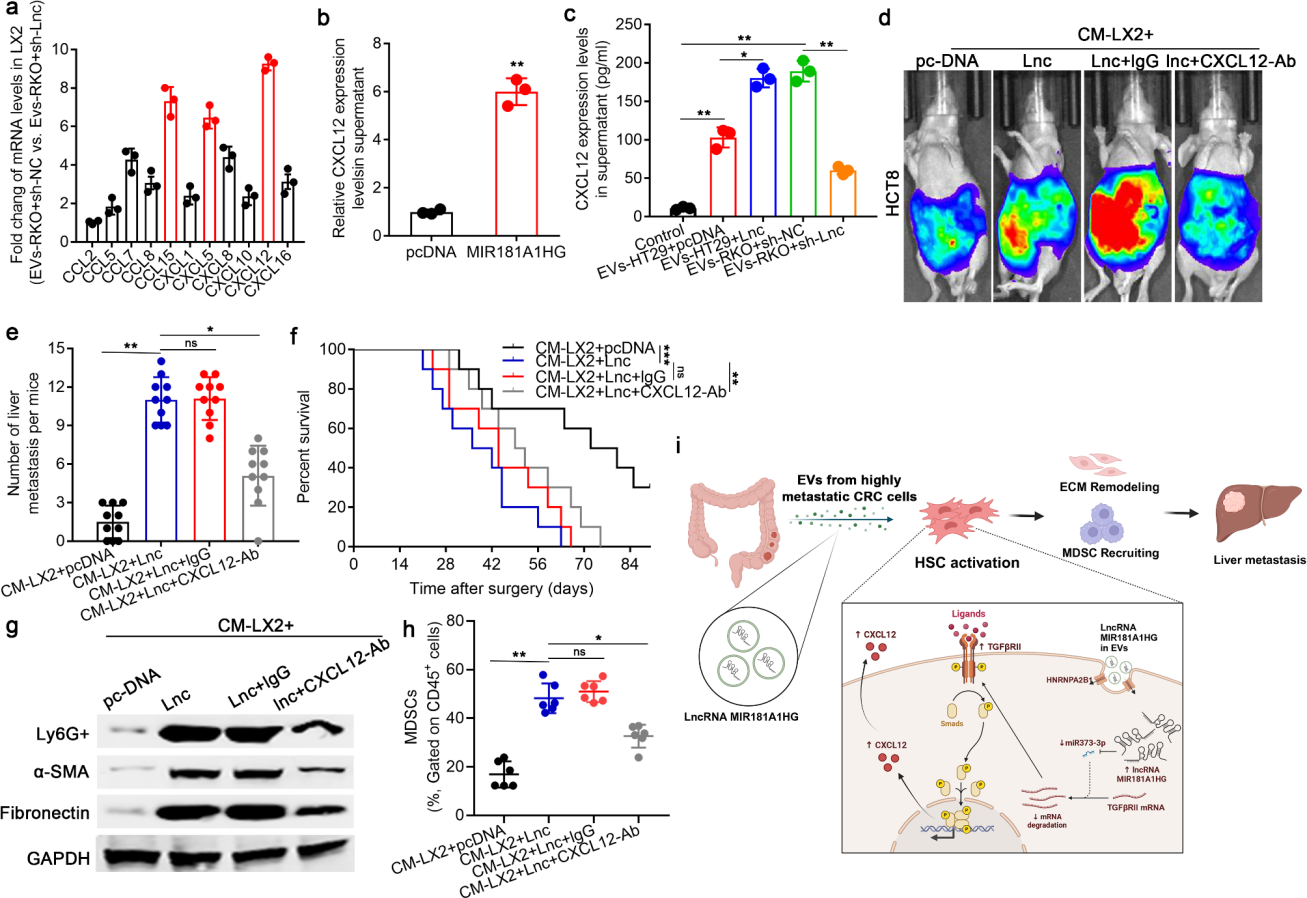
HSCs activated by MIR181A1HG in CRC cell-derived EVs promote CRLM by secreting CXCL12 to induce premetastatic niche formation through recruiting MDSCs and remodeling the ECM in the liver. (**a**) LX2 cells were first activated by coculturing with RKO CRC cells, which were first transfected with MIR181A1HG-knockdown or control constructs. A human chemokine screening test kit was subsequently used to identify differentially expressed chemokines in LX2 cells. (**b**) ELISAs were used to assess the effect of exogenous MIR181A1HG on the expression of CXCL12 in LX2 cells. LX2 cells were first activated by coculturing with EVs derived from HT29 and RKO cells, which had been pretransfected with MIR181A1HG-overexpressing or MIR181A1HG-knockdown constructs. (**c**) The expression of CXCL12 in LX2 cells was determined by ELISA. The luciferase-labeled CRC cell line HCT8 was then injected into the spleens of nude mice to establish a mouse model of CRLM, and conditioned medium (CM) was injected into the tail vein. The impact of CXCL12 secreted by activated HSCs on CRLM in vivo was determined via spleen injection to construct mouse models of CRLM. (**d**) Representative live images of LMs in nude mice. (**e**) Number of metastatic colonies in the livers of the nude mice from different groups determined by live imaging and HE staining. (**f**) Overall survival of each group of mice (*n* = 10 for each group). (**g**) The expression levels of Ly6G+, α-SMA and fibronectin in the LM tissues of each group were assessed by western blotting. (**h**) Quantification of MDSCs in each group of mice. (**i**) Schematic model showing that the lncRNA MIR181A1HG in EVs derived from highly metastatic CRC cells functions as a ceRNA by sponging miR373-3p to activate HSCs via the TGFβRII/Smad2/3 signaling pathway in CRLM. (^ns^*P*>0.05, *P < 0.05, **P < 0.01, ***P < 0.001)

Chemokines, small proinflammatory chemoattractant cytokines, are major regulators of cell trafficking and adhesion that promote cancer progression within the TME [19, 20]. As α-HSCs can secrete cytokines that can stimulate the migration of cancer cells, we speculated that α-HSCs may promote CRLM via the secretion of chemokines. Therefore, using a human chemokine screening test kit, we tested the levels of chemokines in LX2 cells that were precultured with EVs derived from RKO cells with MIR181A1HG knockdown and a negative control group. Among all the assessed chemokines, CCL15, CXCL5, and CXCL12 were the most significantly differentially expressed chemokines (Fig. 6a). Furthermore, when MIR181A1HG was overexpressed in LX2 cells, we also found that the difference in the expression of CXCL12 was considerably greater than that of CCL15 and CXCL5 (Fig. 6b; Supplementary Fig. S8a, b). Next, we cultured LX2 cells with EVs derived from HT29/RKO cells that were pretransfected with MIR181A1HG-overexpressing or anti-MIR181A1HG constructs and then tested the expression levels of CXCL12 by ELISA. As shown in Fig. 6c, overexpression or knockdown of MIR181A1HG partially enhanced or neutralized the promotive effects of HT29/RKO cell-derived EVs on the secretion of CXCL12 in LX2 cells, respectively. To further investigate the role of CXCL12 in CRLM, LX2 cells were first transfected with the MIR181A1HG overexpression construct and treated with an anti-CXCL12 antibody or IgG. Then, we injected HCT8 cells into the spleens of nude mice to construct mouse models of CRLM, and conditioned medium (CM) was injected into the tail vein. The inhibition of CXCL12 partially counteracted the enhancing effect of α-HSCs, which were activated by MIR181A1HG-overexpressing CRC cell-derived EVs, on CRLM (Fig. 6d-f, Supplementary Fig. S8c). Moreover, inhibition of CXCL12 could also partially counteract the ability of MIR181A1HG in CRC cell-derived EVs to recruit MDSCs and remodel the ECM in the liver (Fig. 6g, h). Together, the above results revealed that MIR181A1HG in CRC cell-derived EVs could activate HSCs to promote CRLM via the secretion of CXCL12 in the liver (Fig. 6i).

## Discussion

CRC is the third most common cause of cancer-related death worldwide, and population-based studies have shown that approximately 50% of patients diagnosed with CRC develop LM during the course of their disease [21]. Indications for curative-intended treatment of CRLM have expanded in recent years. Unfortunately, despite the oncological and surgical advances made, only approximately 25% of affected patients are amenable to resection, which is regarded as the only way to achieve a cure [21, 22]. Thus, the development of new treatments directed at the metastatic spread of CRC cells may prove particularly valuable, which underscores the need for further research into the pathogenesis of CRLM. Accordingly, exploration of the mechanism of CRLM and identification of a new strategy for CRLM treatment are urgently needed. In the present study, we demonstrated that the lncRNA MIR181A1HG was progressively upregulated in tissues and serum as well as in EVs from healthy normal controls to CRC patients and paired liver metastatic groups and that a high level of MIR181A1HG in EVs predicted poor survival in CRLM patients. Additionally, we verified that HNRNPA2B1 mediated the packaging of MIR181A1HG into CRC EVs, which was shown to function as a ceRNA by sponging miR373-3p to activate HSCs via the TGFβRII/Smad2/3 signaling pathway. Furthermore, the HSCs activated by MIR181A1HG in CRC cell-derived EVs could secrete the chemokine CXCL12 to promote CRLM by remodeling the ECM and recruiting MDSCs in liver metastases.

Because of the wide landscape of genomic alterations and the limited therapeutic success of CRLM, recent studies have focused on better understanding and possibly targeting the formation of PMNs in the TME [23, 24]. Over the past two decades, it has become evident that the otherwise hostile milieu of the liver is selectively preconditioned at an early stage to render it more conducive to the engraftment and growth of disseminated cancer cells, a concept defined as PMN formation [25]. The development of PMNs is governed by a complex series of reciprocal interactions between tumor cells and various components of the TME, as well as the exploitation of resident and recruited cells in secondary target organs [25, 26]. Growing evidence indicates that tumor-derived EVs serve as mediators of long-distance cell-to-cell communication and play a pivotal role in PMN formation by inducing vascular leakiness, inflammation and bone marrow progenitor cell recruitment [24, 25]. EVs carry molecules such as oncoproteins and oncopeptides, RNA species (microRNA, mRNA, and lncRNA), lipids, and DNA fragments from donor to recipient cells, initiating profound phenotypic changes in the TME, which may be useful not only for predicting metastatic propensity but also for determining organ sites of future metastasis [10, 27]. In fact, we have long investigated the functional mechanism of CRLM and verified that tumor-derived exosomal miR-934 induces macrophage M2 polarization to promote CRLM; [28] miR-181a-5p-rich EVs released from highly metastatic CRC cells promote CRLM by activating HSCs and remodeling the TME [17]. LncRNAs, which are highly enriched in EVs, are involved in a large variety of biological processes, with reports linking the dysregulation of lncRNAs with cancer cell invasion and metastasis through mechanisms including affecting transcriptional and posttranscriptional levels.[24, 25] Accordingly, the effects of lncRNAs in CRC cell-derived EVs on the formation of PMNs during CRLM deserve further exploration.

For successful colonization in the liver, the adaptation of tumor cells and the surrounding stroma is essential [26], and HSCs, which are liver-specific pericytes, function as key players in the response of the liver to invading cancer cells [7]. HSCs are a component of PMNs that transdifferentiate into tumor-promoting myofibroblasts via regulation by TGF-β, a cytokine derived from tumor cells themselves or other stroma cells within the TME [29, 30]. Upon activation, HSCs differentiate into myofibroblast-like cells, leading to increased deposition of the ECM component fibronectin, which is critical for the formation of PMNs in the liver [31]. Moreover, activated HSCs shape an immunosuppressive milieu to protect metastatic tumors against immune surveillance by the production of chemokines, further recruiting MDSCs, which are a heterogeneous population of incompletely matured cells of myeloid origin endowed with potent immunosuppressive activity [32, 33]. In addition, excessive ECM secreted by activated HSCs also forms a physical barrier limiting T-cell mobility and penetration into the tumor area [34]. The TGF-β signaling pathway has been linked to the formation of PMNs and the infiltration of tumor cells because of its key roles in HSC activation, ECM remodeling, and the creation of an immune suppressive environment [26]. TGF-β signaling in the liver stroma can be triggered after the uptake of EVs secreted by the primary tumor [25, 26]. In this context, exploring the mechanisms that regulate TGF-β-induced HSC activation may lead to novel strategies to target hepatic PMNs for CRLM. In the present study, we demonstrated that the lncRNA MIR181A1HG was highly expressed in EVs derived from highly metastatic CRC cells and served as a ceRNA by sponging miR373-3p to activate HSCs via the TGFβRII/Smad2/3 signaling pathway in the liver.

HSCs activated by cancer cells can secrete a plethora of chemokines, which are significantly upregulated in LMs, promoting cancer growth and progression within the TME [19, 20, 29, 35]. During LM in breast cancer, activated HSCs retain and render natural killer cells quiescent, thus suppressing immune surveillance and licensing the re-emergence of disseminated tumor cells through secretion of CXCL12; [20] HSCs play an important role in LM in colon cancer cells by the SDF-1/CXCR4 axis and provide preclinical evidence that blockade of the axis is a target for antimetastasis therapy [19]. In this respect, it would be worth assessing whether HSCs activated by MIR181A1HG in CRC cell-derived EVs secreted chemokines that were involved in the formation of PMNs in CRLM. In our study, we found that CXCL12 expression was apparently upregulated in HSCs activated by MIR181A1HG in CRC cell-derived EVs and that activated HSCs promoted CRLM by secreting CXCL12 to remodel the ECM and recruit MDSCs in liver metastases.

## Conclusions

Our study demonstrated that the lncRNA MIR181A1HG in EVs derived from highly metastatic CRC cells functioned as a ceRNA by sponging miR373-3p to activate HSCs via the TGFβRII/Smad2/3 signaling pathway. Furthermore, activated HSCs primed PMNs in the liver by remodeling the ECM and recruiting MDSCs, ultimately promoting CRLM. Our findings identified MIR181A1HG in highly metastatic CRC cell-derived EVs as a prognostic factor for CRLM and a promoter of PMN formation in the liver. Targeting MIR181A1HG in CRC cell-derived EVs or its regulated signaling pathway might provide a novel strategy for curtailing the prometastatic host response of the liver during the early stages of CRLM.

## Electronic supplementary material

Below is the link to the electronic supplementary material.

Supplementary Material 1

Supplementary Material 2

## Data Availability

Not applicable.
