# Supplementary material for: The lncRNA MIR181A1HG in extracellular vesicles derived from highly metastatic colorectal cancer cells promotes liver metastasis by remodeling the extracellular matrix and recruiting myeloid-derived suppressor cells
Source: Cell Biosci. 2025 Feb 19;15:23. doi: 10.1186/s13578-025-01365-2 (PMC11841002; doi:10.1186/s13578-025-01365-2)

Fig. 2h：

a-SMA：

Ly6G+：

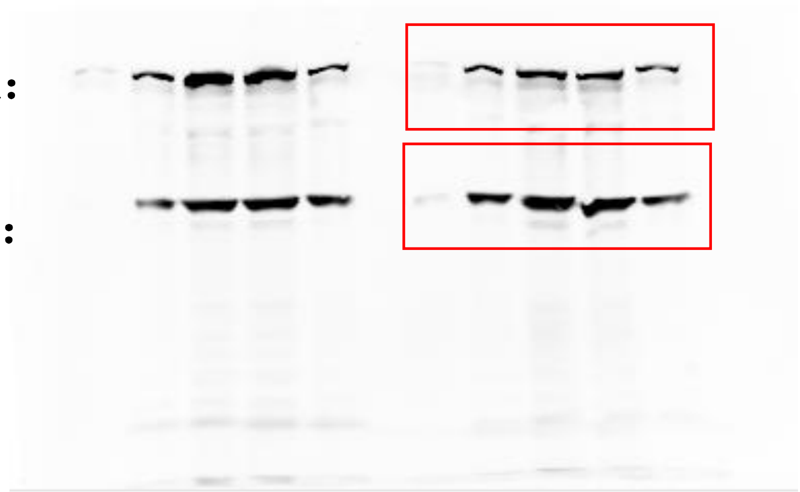

Fibronectin：

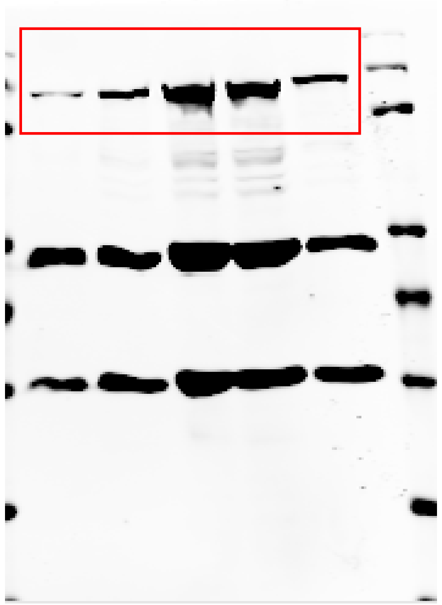

GAPDH：

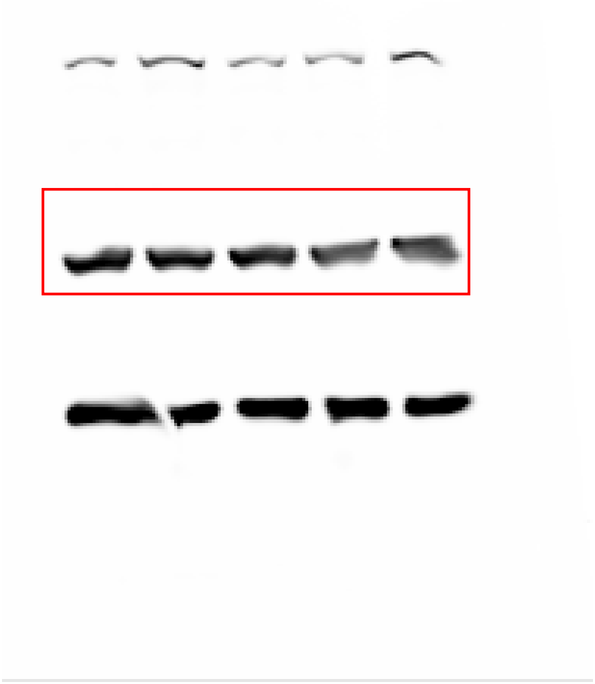

Fig. 3g

HNRNPA2B1：

Controls：

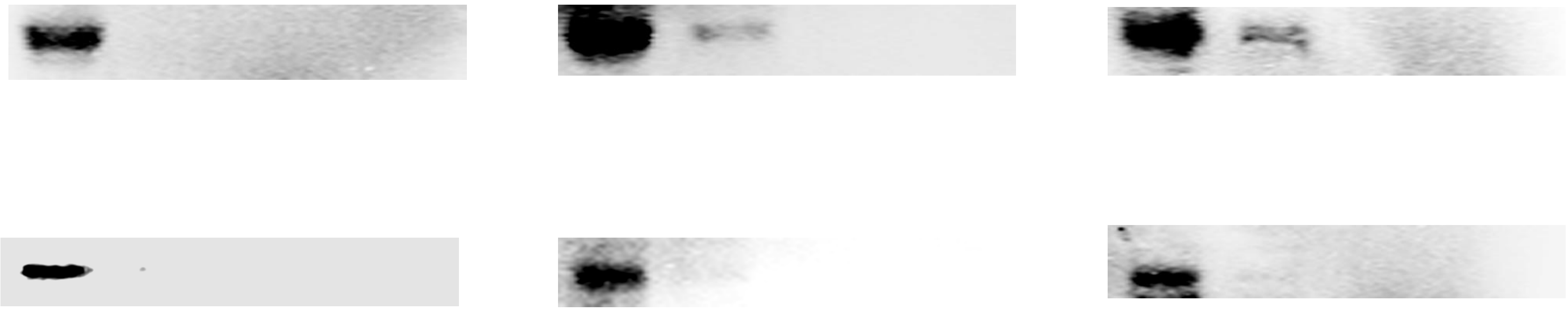

Fig. 3h

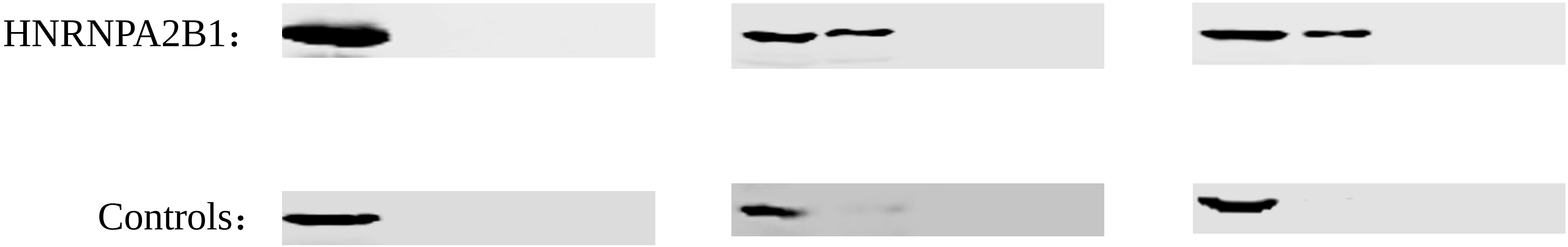

Fig. 4l

TGFBRII:

GAPDH:

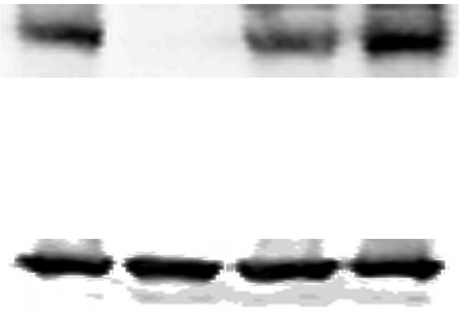

Fig. 4p

TGFBRII：

GAPDH：

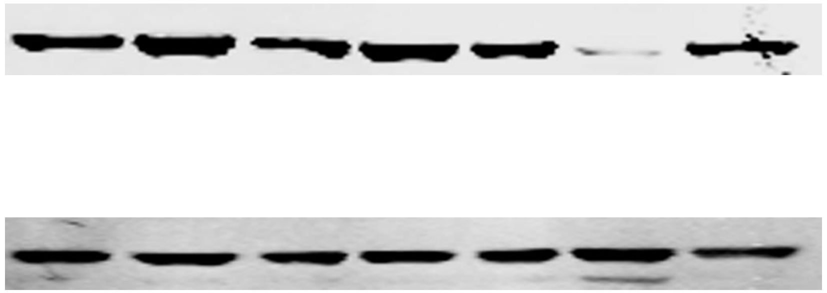

Fig. 4q

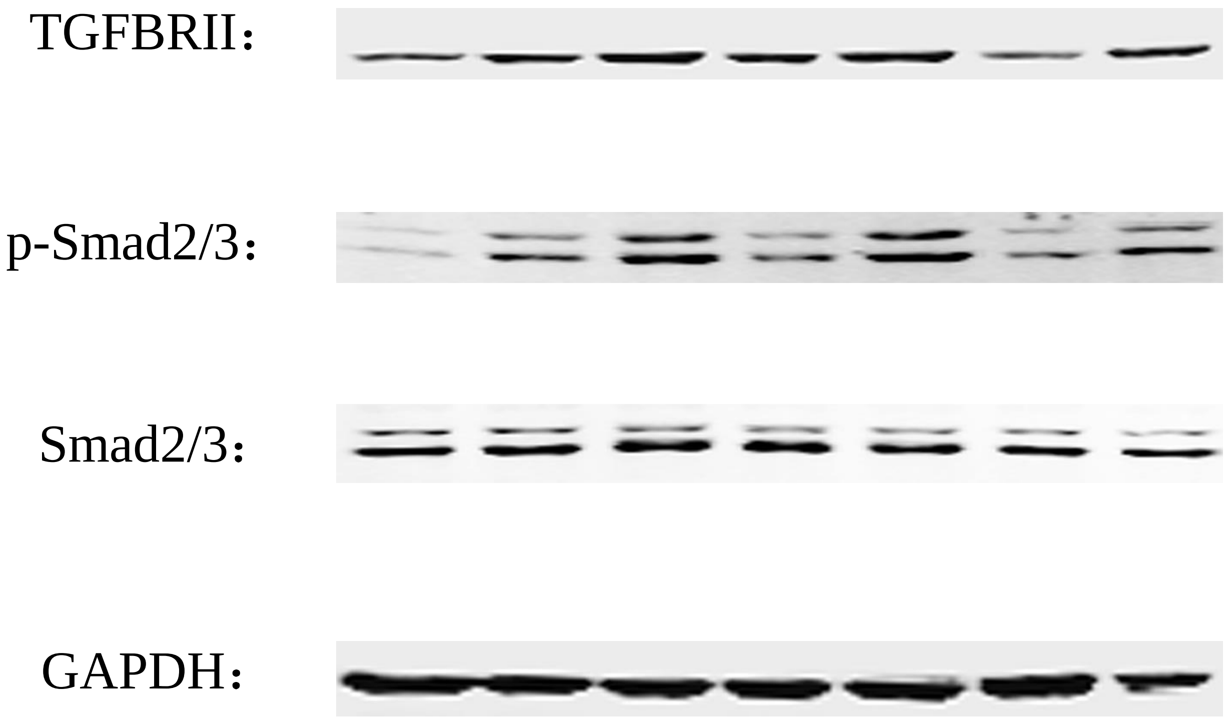

Fig. 4r

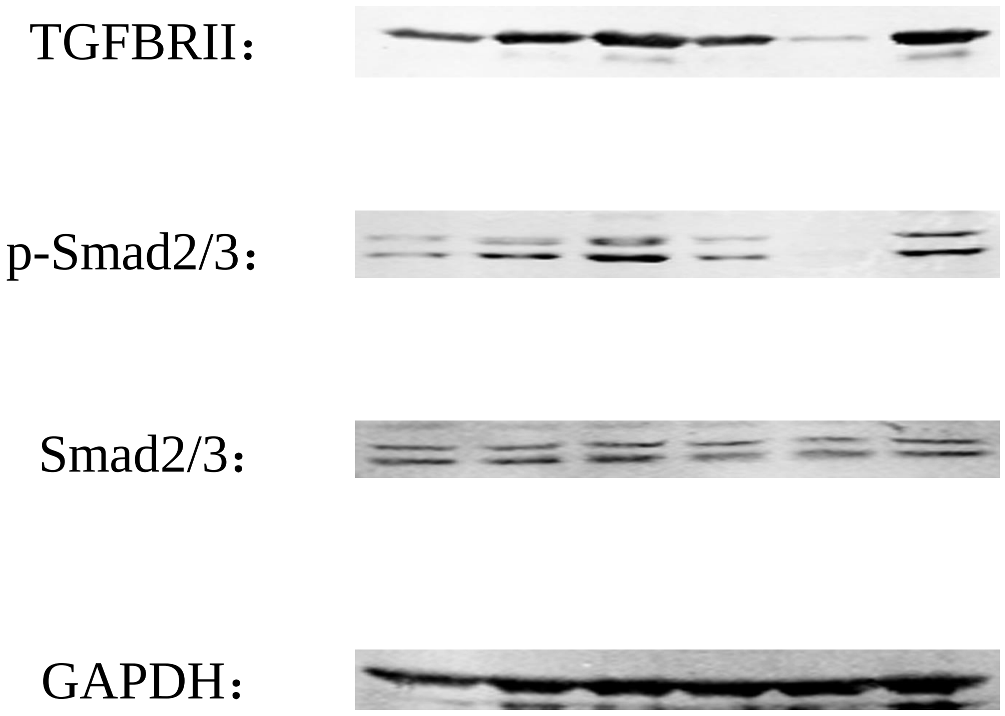

Fig. 4s

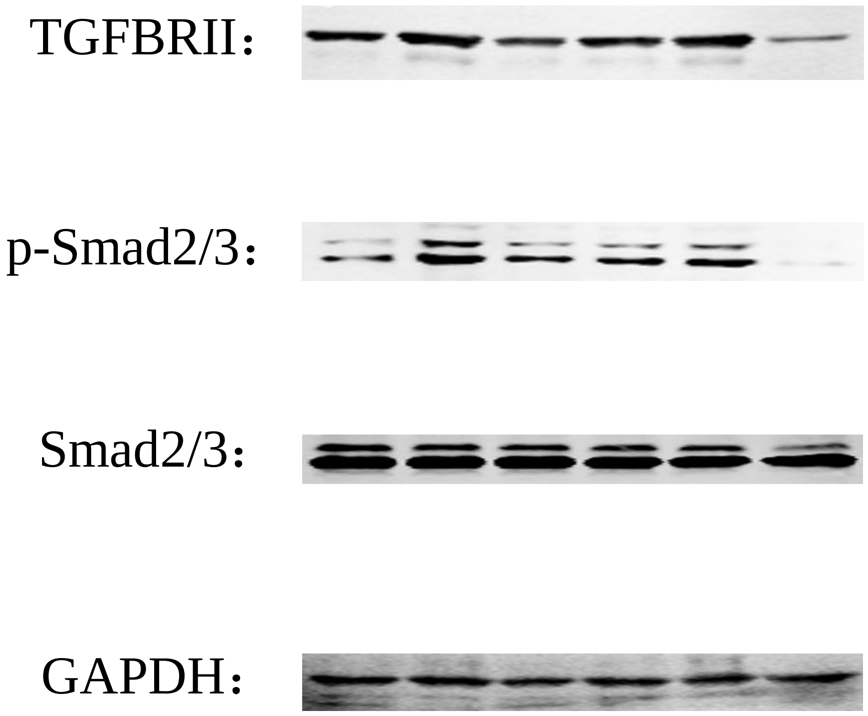

Fig. 5g

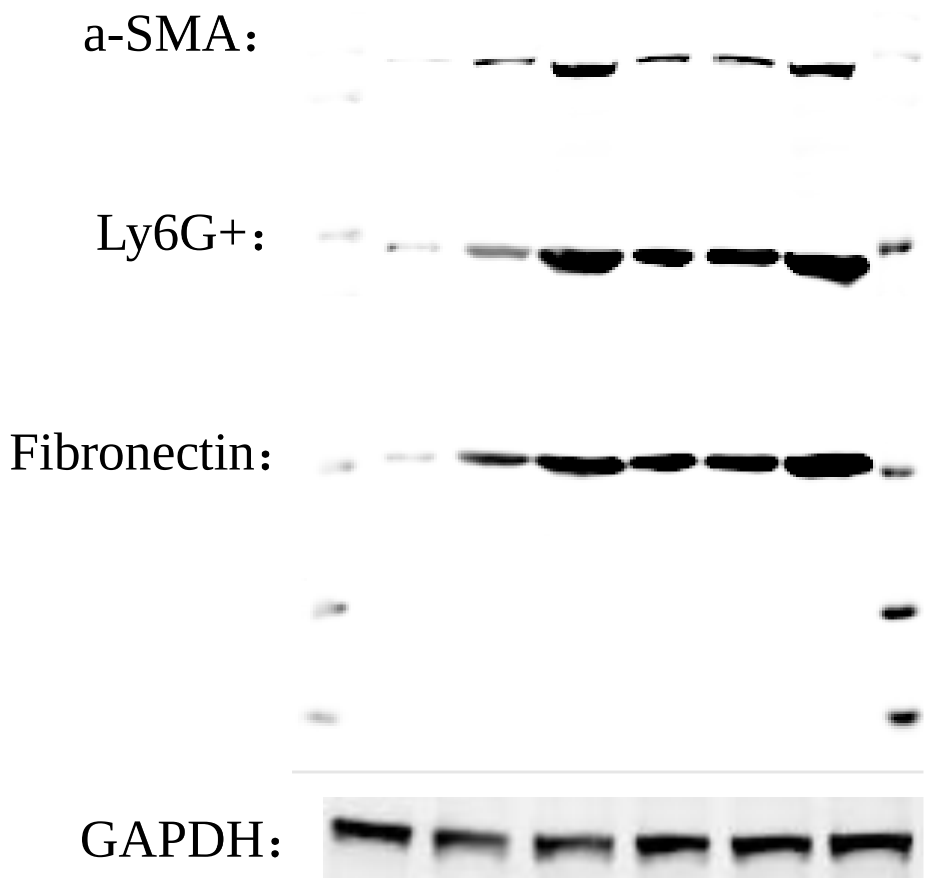

Fig. 5

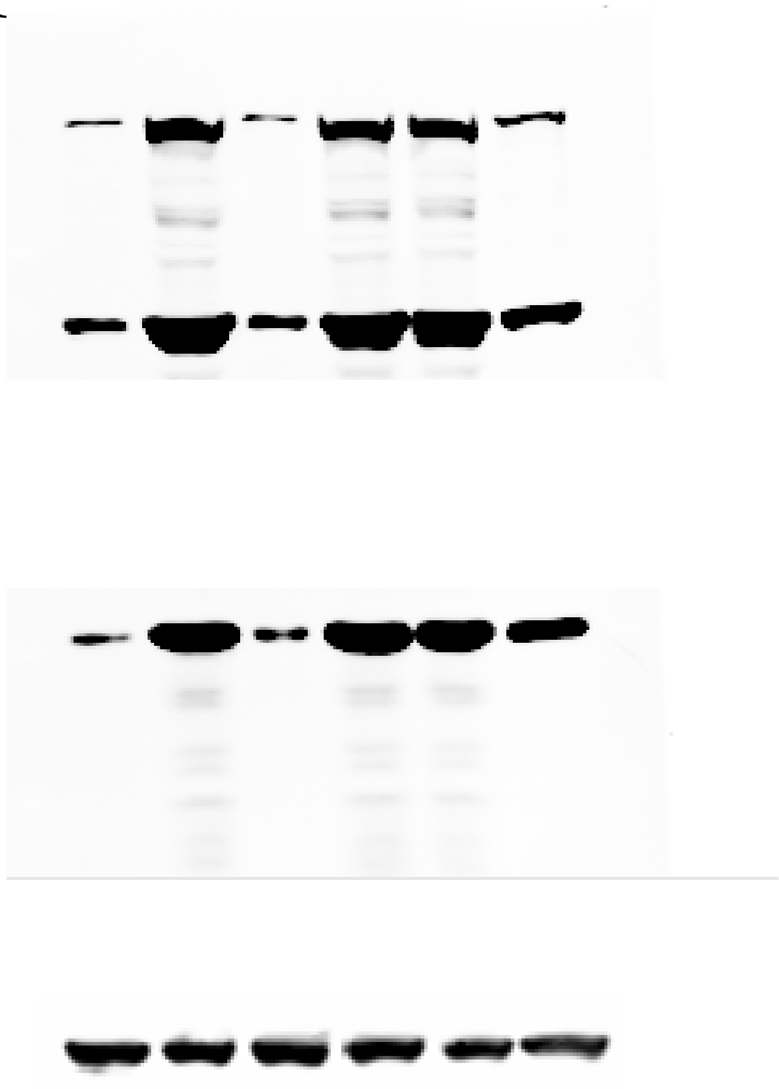

Fig. 6g

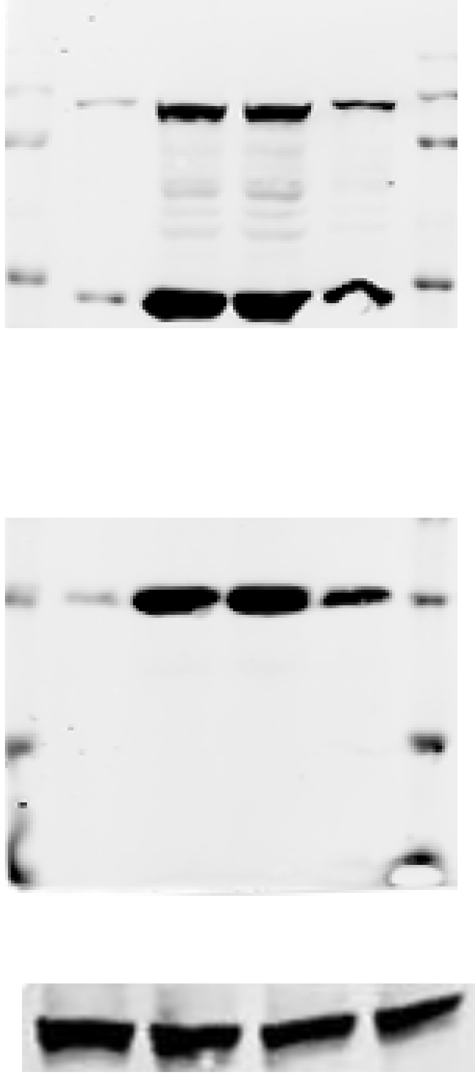

a-SMA:

Ly6G+:

Fibronectin:

GAPDH:

Fig. S3e

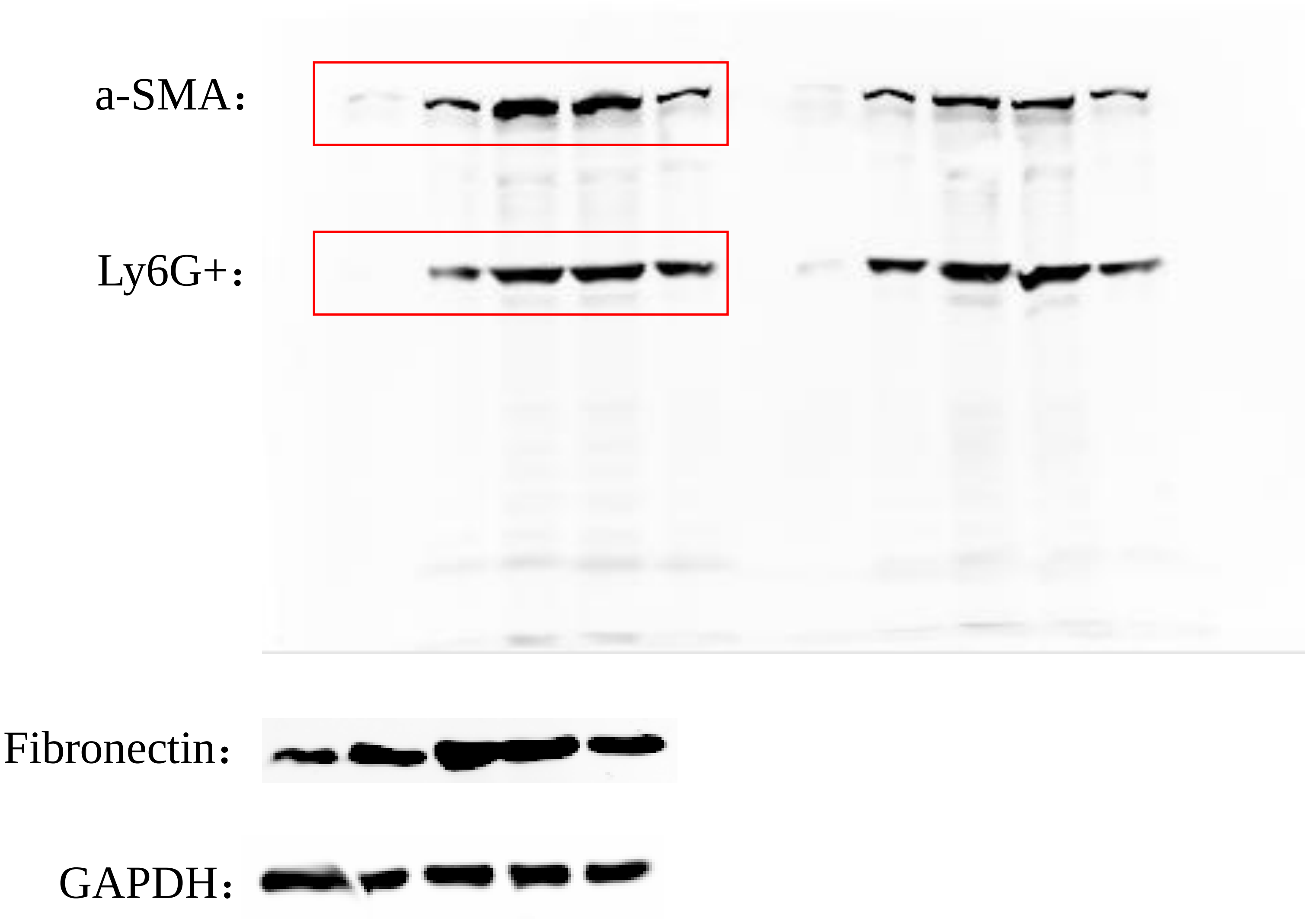

Fig. S4a

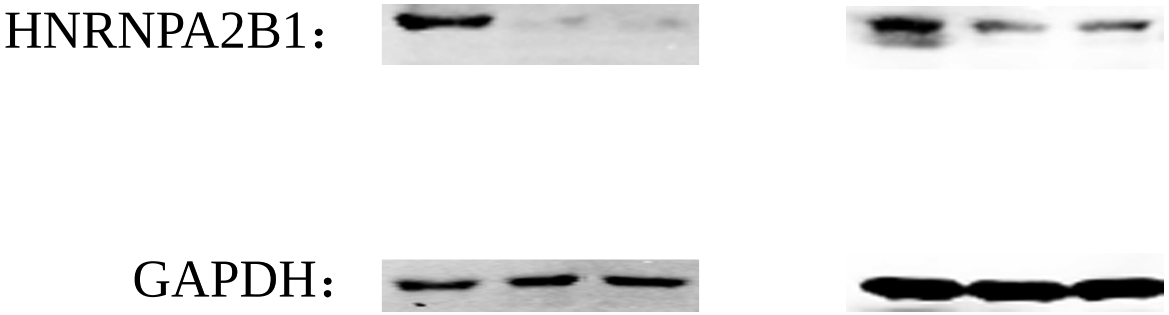

Fig. S4b

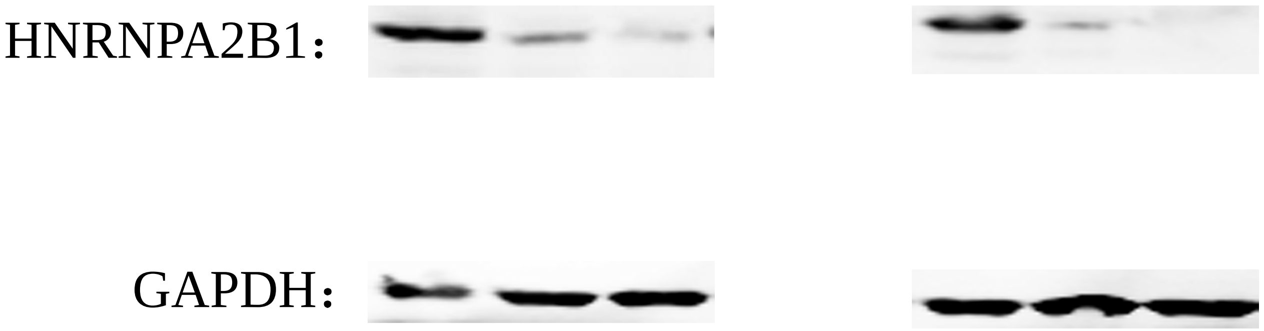

Fig. S4c

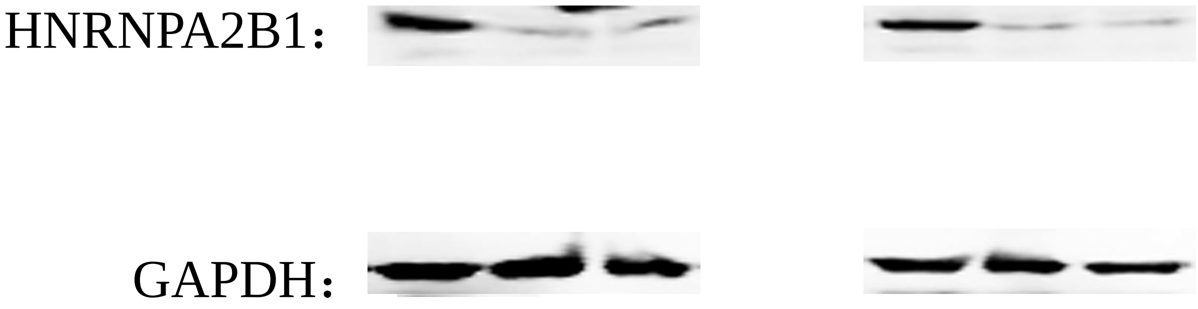

Supplement: Supplementary file 1 — Supplementary Material 1 [file 13578_2025_1365_MOESM1_ESM.pdf]
