# Supplementary material for: The lncRNA MIR181A1HG in extracellular vesicles derived from highly metastatic colorectal cancer cells promotes liver metastasis by remodeling the extracellular matrix and recruiting myeloid-derived suppressor cells
Source: Cell Biosci. 2025 Feb 19;15:23. doi: 10.1186/s13578-025-01365-2 (PMC11841002; doi:10.1186/s13578-025-01365-2)
Supplement: Supplementary file 2 — Supplementary Material 2 [file 13578_2025_1365_MOESM2_ESM.docx]

**Supplementary materials and methods:**

**Cell culture**

The human CRC cell lines HT29, RKO, SW480, SW620, and HCT8 and the HSC line LX2 were purchased from the Cell Bank of Type Culture Collection of the Chinese Academy of Sciences (Shanghai, China). All the cells were cultured in Dulbecco’s modified Eagle’s medium (Gibco; Thermo Fisher Scientific, USA) supplemented with 10% fetal bovine serum (Invitrogen; Thermo Fisher Scientific, USA) in a humidified atmosphere containing 5% CO_2_ at 37 °C. Cell transfection was performed with Lipofectamine 2000 according to the manufacturer’s instructions.

**Fluorescent labeling and tracing of MIR181A1HG in EVs**

The CRC cell lines RKO and SW620 were pretreated with Cy3-labeled MIR181A1HG. The CRC cells were subsequently cocultured with LX2 cells for 48 h in 24-well Transwell chambers. Finally, the degree of EV-encapsulated MIR181A1HG internalization was examined with a confocal laser-scanning microscope (TCS SP8; Leica Microsystems GmbH, Germany). The nuclei of LX2 cells were stained with 4′,6-diamidino-2-phenylindole (DAPI; Roche Diagnostics, Switzerland).

**RNA extraction and quantitative PCR (qPCR) assay**

Total RNA from cells was extracted with TRIzol^®^ reagent (Takara Bio, Japan), and RNA extraction from EVs was performed with SeraMir Exosome RNA Purification for Media & Urine (cat. no. RA806TC-1; System Biosciences) according to the manufacturer’s instructions. After the RNA intensity and purity were assessed, 2 μg of total RNA was reverse transcribed into cDNA with the RevertAid™ First Strand cDNA Synthesis Kit (Fermentas, USA) according to the manufacturer’s instructions. Then, 1 μg of cDNA was used as a template for qPCR with SYBR Premix Ex Taq (Takara, Japan) according to the manufacturer’s instructions. The amplification protocol used was as follows: an initial denaturation step for 2 min at 95 °C followed by 40 cycles of denaturation for 10 s at 95 °C, annealing for 30 s at 62 °C, elongation for 30 s at 72 °C, and a final extension step at 72 °C for 30 s. Relative quantities (Δ cycle threshold (Ct) values) were obtained by normalization to the levels of GAPDH and U6. All sequences of primers used in this study are summarized in Supplementary Table S2.

**Western blot (WB)** **assay**

The proteins were resolved by 10% sodium dodecyl sulfate‒polyacrylamide gel electrophoresis (SDS‒PAGE) and then transferred onto PVDF membranes (Millipore Sigma, USA) by standard protocols. The blots were incubated with primary antibody overnight at 4 °C. Following three washes, the membranes were then incubated with secondary antibody for 2 h at room temperature, and signals were visualized with an enhanced chemiluminescence (ECL) reagent (Millipore, Sigma, USA). The antibodies used in the present study are summarized in Supplementary Table S3.

**Hematoxylin‒eosin (HE) staining**

The paraffin-embedded tissues were dewaxed with xylene, anhydrous ethanol, ethanol and distilled water successively. Then, the cell nuclei were stained with hematoxylin, and the cytoplasm was stained with eosin. The slices were subsequently dehydrated and cleared through incubation with alcohol, anhydrous ethanol and xylene and sealed with neutral glue. Finally, the slices were observed via microscopic examination, and images were collected and analyzed.

**Cell migration and invasion assays**

The migration and invasion abilities of CRC cells were evaluated by using 24-well Boyden chambers (Corning, USA), and an 8-μm pore polycarbonate membrane was used to assess cell invasion according to the manufacturer’s protocol. Briefly, each group of cells (5x10^4^ cells/chamber) in serum-free medium was seeded in the upper chambers, while the bottom chambers were filled with 600 μl of basal medium containing 10% FBS. The migrated or invaded cells in the bottom chamber were fixed with methyl alcohol and stained with 0.1% crystal violet solution. The stained cells were counted under a microscope in four randomly selected files, and the experiment was performed in triplicate.

***In vivo* metastasis experiments**

To explore the impact of EVs derived from CRC cells on CRLM *in vivo*, 1x10^6^ luciferase-labeled CRC cells were injected into the spleens of male BALB/c nude or C57BL/6 mice for 4 weeks. There were 10 mice in each group, and within 1 month, the mice in each group were injected twice with equal quantities of EVs (30 μg) derived from CRC cells. Tumor metastases that had formed in the livers were observed and macroscopically examined for gross lesions and counted after HE staining under a microscope. All animals received humane care according to the criteria outlined in the [Guide for the Care and Use of Laboratory Animals](https://wol-prod-cdn.literatumonline.com/pb-assets/hub-assets/aasldpubs/Hepatology_1527-3350/guide-for-the-care-and-use-of-laboratory-animals-1542041308777.pdf) prepared by the National Academy of Sciences, and the animal assays were approved by the Institutional Animal Care and Use Committee of Fudan University Shanghai Cancer Center (Shanghai, China).

**Immunofluorescence (IF) staining**

Liver metastasis sections from *in vivo* experiments were fixed with cold acetone, blocked with 20% FBS in PBS and stained with primary antibodies. The cells were first washed with PBS and fixed with 4% paraformaldehyde, permeabilized with 0.1% sodium citrate plus 0.1% Triton X-100, and stained with primary antibodies overnight at 4 °C. After being washed with cold PBS three times, the cells were incubated with a fluorescence-labeled secondary antibody and finally visualized with the Nikon confocal microscope A1R with appropriate lasers.

**Flow cytometry**

The liver tissues were harvested after draining the blood, rinsed thoroughly in PBS and minced into small pieces (< 0.5 × 0.5 mm in size) in DMEM. The tissues were then incubated with intermittent agitation, filtered through a 70-μm nylon strainer (Corning, USA) and washed with DMEM. After red blood cells were removed by resuspension in ACK lysis buffer (Life Technologies, Thermo Fisher Scientific, USA), the cells were counted and stained with an Aqua dead cell stain kit (Life Technologies, Thermo Fisher Scientific, USA) according to the manufacturer’s protocol. Then, the cells were washed with PBS containing 0.2 mM EDTA with 2% FBS and stained with appropriate antibodies. Finally, the cells were washed with PBS containing 0.2 mM EDTA with 2% FBS and examined with a FACSCanto II (BD Biosciences, USA). Flow cytometric data were analyzed with FlowJo (FlowJo, LLC, version 10.2).

**Luciferase reporter assay**

To determine the binding sites among MIR181A1HG, miR-373-3p and TGFβRⅡ in LX2 cells, cells were pretransfected with MIR181A1HG-overexpressing or -knockdown vectors and miR-181a-5p, as well as their control vectors. First, LX2 cells were transfected with luciferase constructs containing the wild-type and mutant versions of the binding site. Then, 48 h after transfection, the firefly and Renilla luciferase activities were measured, and the Renilla luciferase activity was used to normalize the transfection efficiency. The results are expressed as relative luciferase activity, which was assessed by a Dual-Luciferase Reporter Assay protocol with a Veritas™ 96-well Microplate Luminometer and a substrate dispenser (Promega Corporation, USA).

**Biotin lncRNA pull-down assay**

First, biotin-labeled wild-type/mutant MIR181A1HG, which were synthesized by Shanghai GenePharma Co., Ltd. (Shanghai, China), were transfected into RKO and SW620 CRC cells for 48 h. Cytoplasmic and nuclear extracts were acquired with NE-PER™ nuclear and cytoplasmic extraction reagents (Thermo Fisher Scientific, USA), respectively, according to the manufacturer's instructions. Then, the nuclear, cytoplasmic or EV lysates were incubated overnight at 4 °C, followed by incubation with M-280 streptavidin magnetic beads (Sigma‒Aldrich, USA). Finally, the precipitates were washed and assessed by WB.

**RNA-binding protein immunoprecipitation (RIP) assay**

The cells were cotransfected with pMS2bp-GFP and MS2, MS2-MIR181A1HG, or MS2-MIR181A1HG mut. After 48 h, the RIP assay was performed with a Magna RIP™ Kit (Millipore, Sigma, USA) according to the manufacturer's instructions. The cell lysates were incubated with antibodies against GFP and IgG. After reverse transcription, the MIR181A1HG level was analyzed by qPCR. For anti-AGO2 RIP, cells were transfected with miR-373-3p mimics, anti-miR-373-3p or controls for 48 h. Then, RIP assays were performed using an anti-AGO2 antibody. qPCR was performed to estimate the level of MIR181A1HG in the RIP products. Fold changes in lncRNA enrichment in the immunoprecipitated samples are presented as a percentage of the input.

**Statistical analysis**

Quantitative data for two or more than two groups were analyzed with Student’s t test or one-way ANOVA, respectively. Qualitative data were analyzed by the chi-square test or Fisher's exact test. Survival curves were plotted with the Kaplan‒Meier method, and differences in survival were compared with a log-rank test. All the statistical analyses were performed with SPSS version 22.0 (IBM Corp., NY, USA). All the experiments were repeated three times, and the data are presented as the means ± standard deviations (SDs). For all tests, *P*<0.05 was considered to indicate statistically significant differences.

**Supplementary Figures and Figure legends:**

**
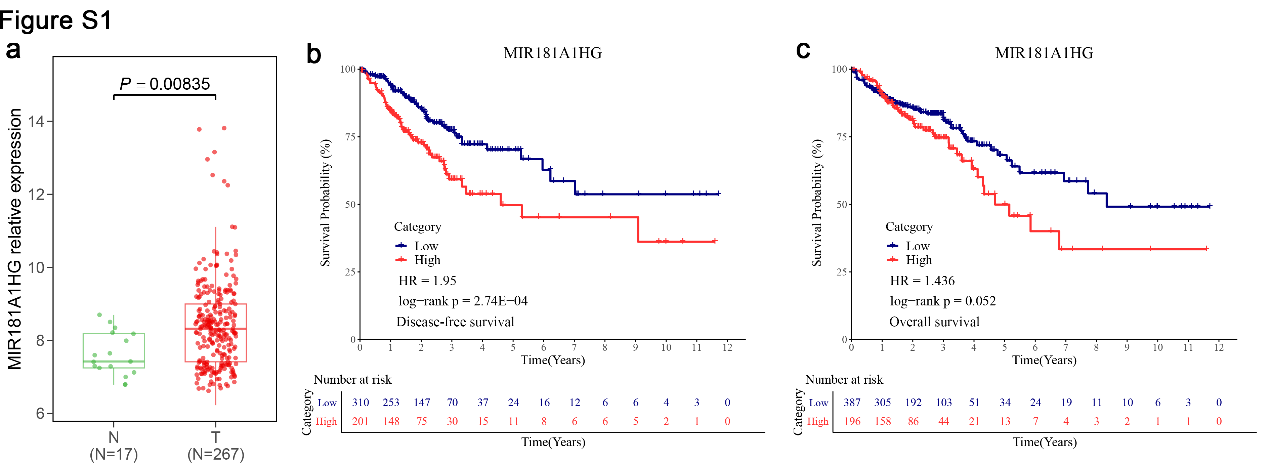
**

**Figure S1.** The expression levels of MIR181A1HG **(a)** and its correlation with disease-free survival **(b)** and overall survival **(c)** in a public database.

**
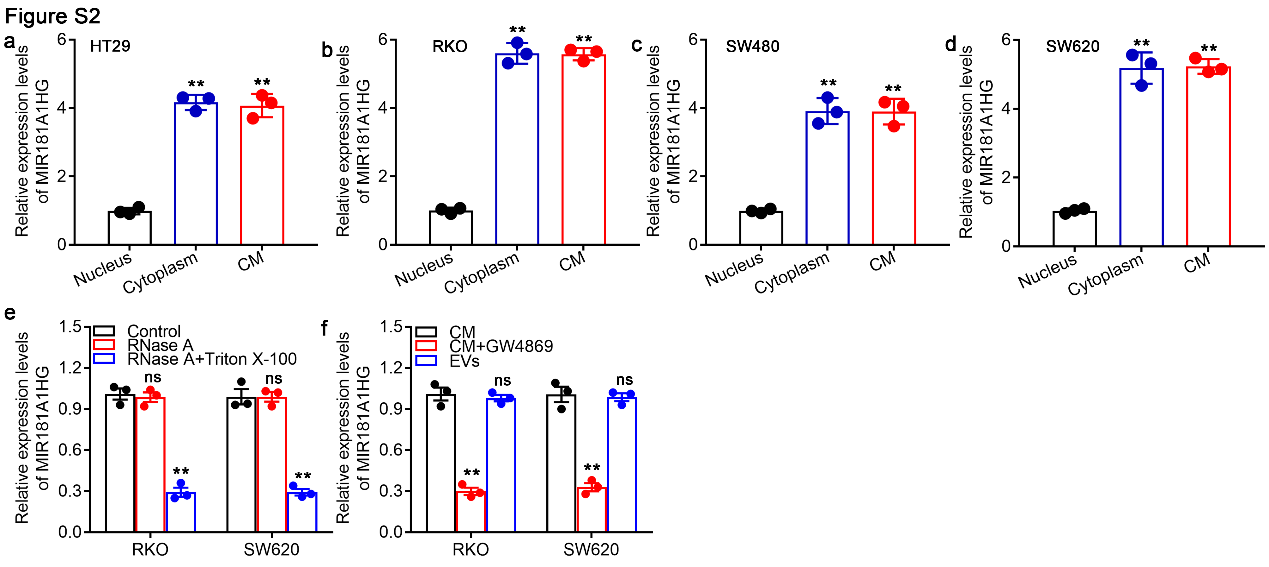
**

**Figure S2.** The expression levels of MIR181A1HG in the nucleus, cytoplasm and culture medium (CM) of HT29 **(a)**, RKO **(b),** SW480 **(c)** and SW620 **(d)** CRC cells were examined by qPCR. **e.** qPCR analysis of the expression levels of MIR181A1HG in RKO/SW620 CRC cells treated with control medium and RNase A (2 mg/ml) alone or combined with Triton X-100 (0.1%) for 0.5 h. **f.** qPCR analysis of the expression levels of MIR181A1HG in CM from RKO and SW620 CRC cells and EVs derived from RKO and SW620 cells untreated or treated with an inhibitor of exosome secretion (GW4869). (^ns^*P*<0.05, ^**^*P*<0.01)

**
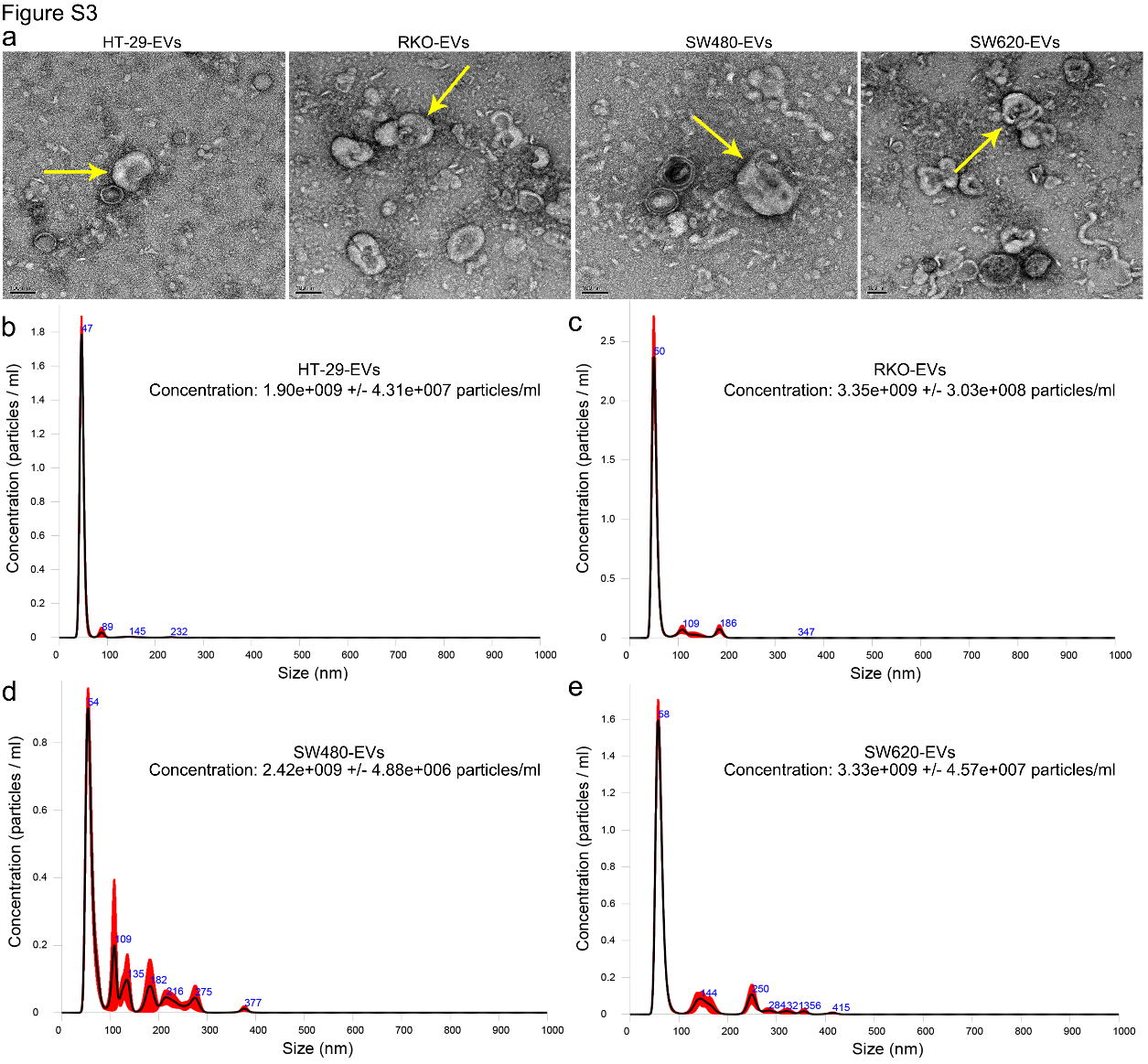
**

**Figure S3. a.** The phenotypes of EVs derived from two weakly metastatic CRC cell lines (HT29 and SW480) and two highly metastatic CRC cell lines (RKO and SW620) were analyzed by electron microscopy; yellow arrows indicate representative EVs. Nanoparticle tracking analysis of EVs derived from the CRC cell lines HT29 (**b**), RKO (**c**), SW480 (**d**) and SW620 (**e**) via NanoSight.

**
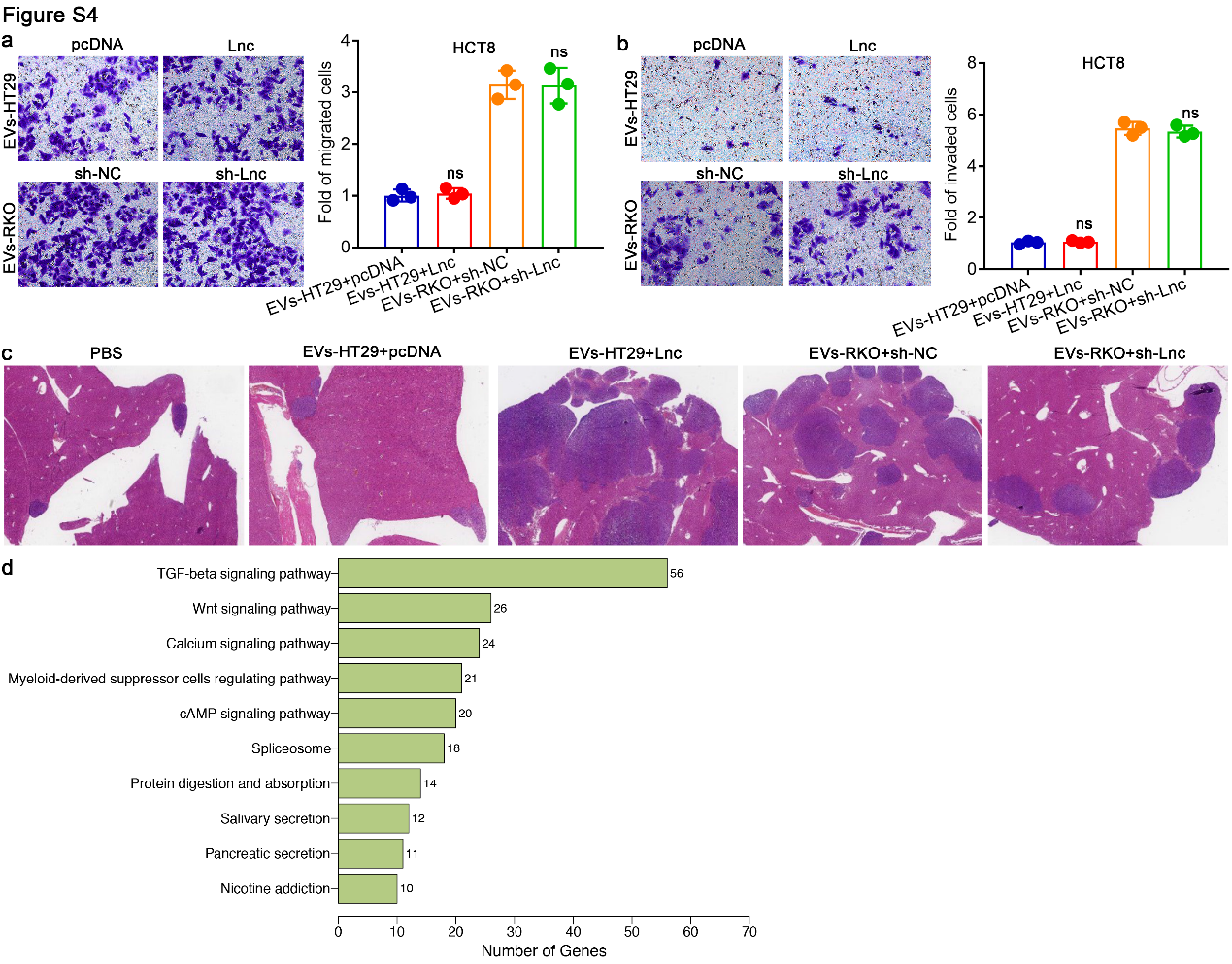
**

**Figure S4.** The CRC cell lines HT29/RKO were transfected with MIR181A1HG overexpression or knockdown constructs, and then EVs were extracted and cocultured with another CRC cell line, HCT8. The effects of EVs derived from the CRC cell lines HT29 (**a**) and RKO (**b**) on CRLM *in vitro* were determined by Transwell assays. **c.** Number of metastatic colonies in the livers of the nude mice from different groups determined by HE staining. **d.** Gene Ontology/Kyoto Encyclopedia of Genes and Genomes (GO/KEGG) enrichment analyses were performed, and the results revealed that the downregulation of MIR181A1HG was significantly associated with myeloid-derived suppressor cell (MDSC) accumulation, extracellular matrix (ECM) deposition and the TGFβ signaling pathway. (^ns^ *P*<0.05)

**
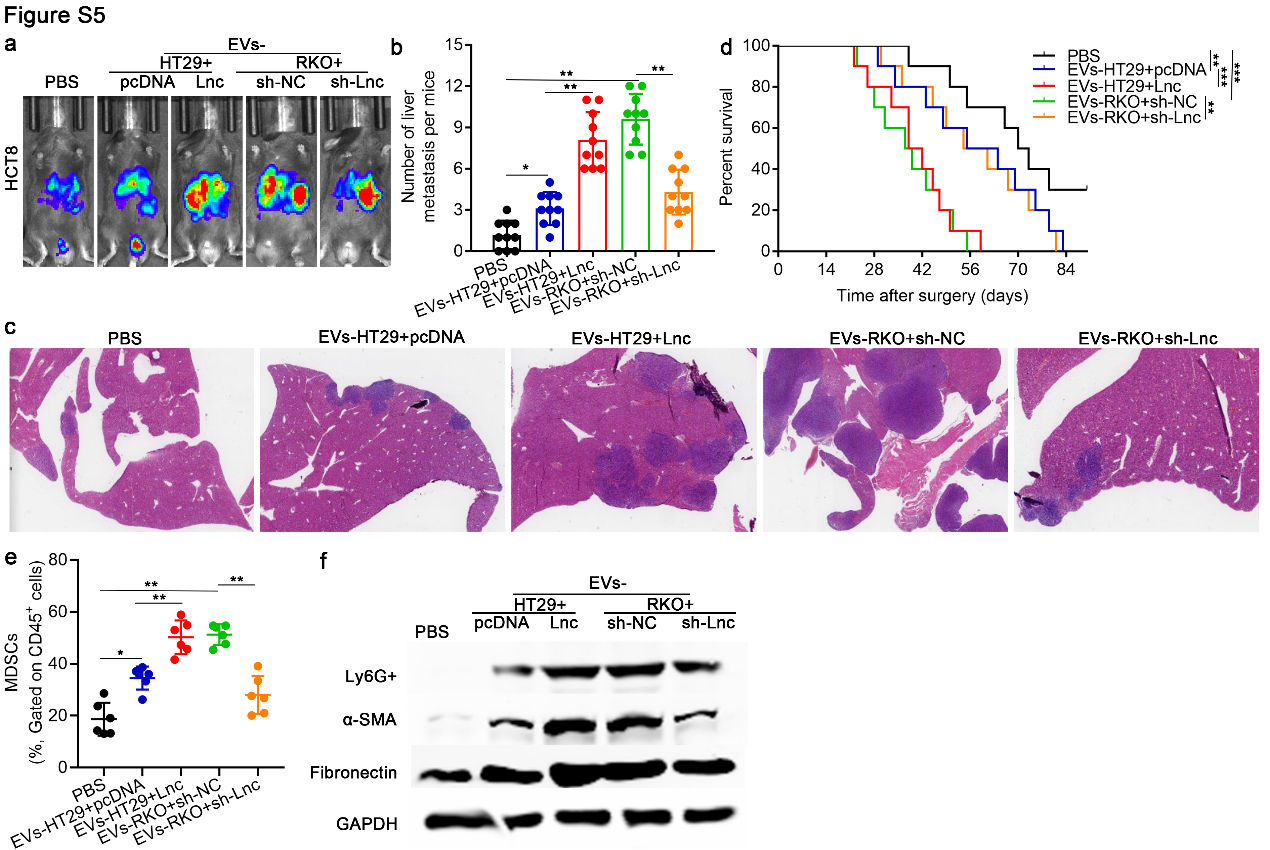
**

**Figure S5.** The CRC cell lines HT29/RKO were transfected with MIR181A1HG overexpression or knockdown constructs, and then EVs were extracted and cocultured with one luciferase-labeled CRC cell line, HCT8. The effects of EVs derived from CRC cells on CRLM *in vivo* were further determined via spleen injection to construct mouse models of CRLM. **a.** Representative live images of liver metastases in C57BL/6J mice. **b.** Number of metastatic colonies in the livers of C57/BL6 mice from different groups determined by live imaging and HE staining (**c**). **d.** Overall survival of each group of mice (n=10 for each group). **e.** Quantification of MDSCs in each group of mice. **f.** The expression levels of Ly6G+, α-SMA and fibronectin in the LM tissues of each group were detected by western blotting. (**P*<0.05, ***P*<0.01, ****P*<0.001)

**
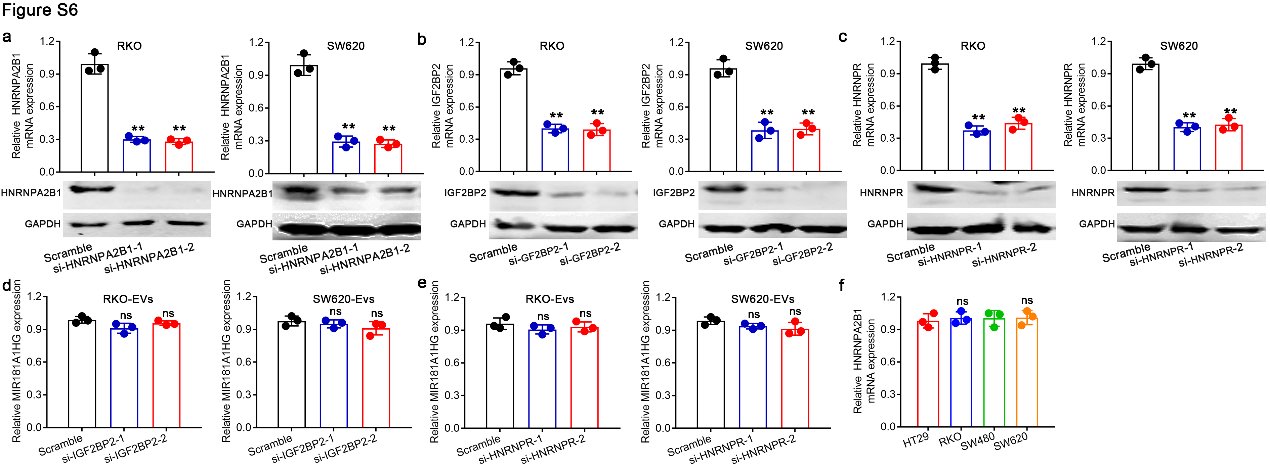
**

**Figure S6.** qPCR and western blotting were used to validate the expression levels of HNRNPA2B1 **(a)**, IGF2BP2 **(b)** and HNRNPR **(c)** in the CRC cell lines RKO and SW620, which were transfected with their specific siRNAs and control vectors. qPCR analysis of the expression levels of MIR181A1HG in EVs derived from the CRC cell lines RKO and SW620 upon IGF2BP2 knockdown **(d)** and HNRNPR knockdown **(e)**. **f.** qPCR analysis of the expression levels of HNRNPA2B1 in the CRC cell lines HT29, RKO, SW480, and SW620. (^ns^*P*>0.05, ***P*<0.01)

**
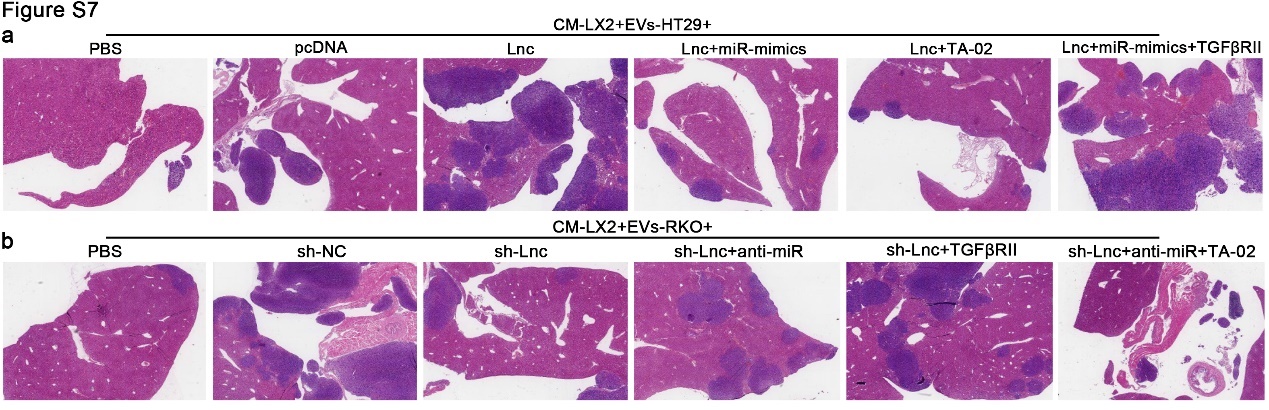
**

**Figure S7.** LX2 cells were first activated by coculturing with EVs derived from HT29 and RKO cells, which had been pretransfected with MIR181A1HG-overexpressing or MIR181A1HG-knockdown constructs. Then, LX2 cells were transfected with miR373-3p mimics or anti-miR373-3p and treated with exogenous TGFβRⅡ or TA-02, respectively. Then, the luciferase-labeled CRC cell line HCT8 was injected into the spleens of nude mice to establish a mouse model of CRLM, and conditioned medium (CM) from activated LX2 cells was injected into the tail vein. **a, b.** Number of metastatic colonies in the livers of nude mice from different groups determined by HE staining.

**
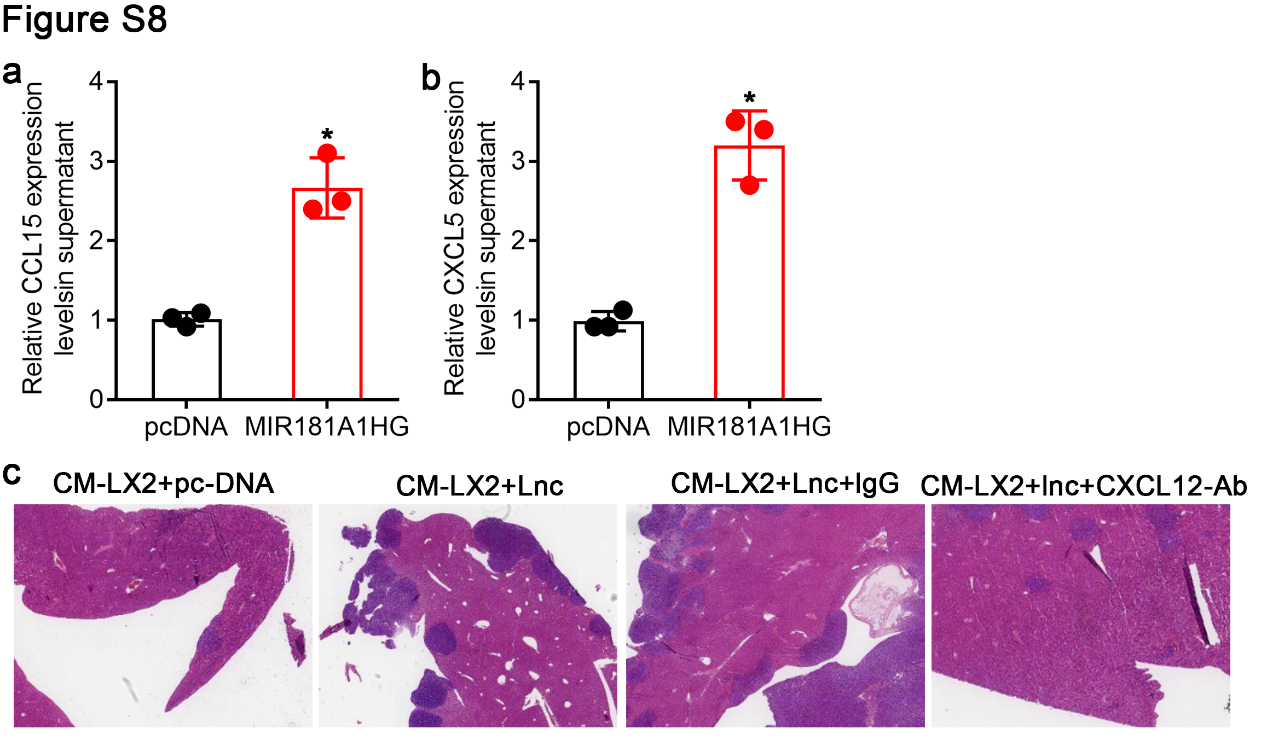
 Figure S8.** **a**. ELISAs were used to assess the effect of exogenous MIR181A1HG on the expression of CCL15 in LX2 cells. **b.** ELISAs were used to assess the effect of exogenous MIR181A1HG on the expression of CXCL5 in LX2 cells. **c.** Number of metastatic colonies in the livers of the nude mice from different groups determined by HE staining. (**P*<0.05)

**Supplementary Table S1: Clinicopathological characteristics of 90 CRC patients.**

| Variable | N |
| --- | --- |
|  | 90 |
| Age (yr) |  |
| <57 | 44 |
| >=57 | 46 |
| Gender |  |
| Male | 52 |
| Female | 38 |
| Tumor location |  |
| Right | 49 |
| Transverse | 19 |
| Left | 3 |
| Sigmoid  Rectum | 3  16 |
| T classification |  |
| T 1 | 1 |
| T 2 | 18 |
| T 3 | 9 |
| T 4 | 62 |
| N classification |  |
| N 0 | 33 |
| N 1 | 28 |
| N 2 | 29 |
| M classification |  |
| M 0 | 51 |
| M 1 | 39 |
| AJCC stage |  |
| I | 16 |
| II | 12 |
| III | 23 |
| IV | 39 |
| Differentiation |  |
| Well | 3 |
| Moderate | 67 |
| Poor | 20 |

**Supplementary Table S2: Data of sequences for qPCR and cell transfection in this study.**

| Gene | Sequence |
| --- | --- |
| MIR181A1HG | Forward: GGCATGATTAAGGTCTCGC  Reverse: TGGAAGGTTGAGTCTGCTG |
| HNRNPA2B1 | Forward: TTACTGATAGGCAGTCTGGA  Reverse: GGTATGGTATTTCTGCAATACG |
| IGF2BP2 | Forward: AGCTAAGCGGGCATCAGTTTG  Reverse: CCGCAGCGGGAAATCAATCT |
| HNRNPR | Forward: GCAAGGTGCAAGAGTCCACA  Reverse: CACGCCAGAGTACACACTGTC |
| miR373-3p | Forward: GAAGUGCUUCGAUUUUGGGGUGU  Reverse: ACCCCAAAAUCGAAGCACUUCUU |
| TGFβRⅡ | Forward: AATCCTGCATGAGCAACTG  Reverse: CATTCTTTCTCCATACAGCCA |
| GAPDH | Forward: ACAACTTTGGTATCGTGGAAGG  Reverse: GCCATCACGCCACAGTTTC |
| U6 | Forward: GCTTCGGCAGCACATATACTAAAAT  Reverse: CGCTTCACGAATTTGCGTGTCAT |
| anti-MIR181A1HG | Target sequences: GCTGAGTTCCGAGTGACTTTA |
| si-HNRNPA2B1-1 | Target sequences: GGAGAGTAGTTGAGCCAAA |
| si- HNRNPA2B1-2 | Target sequences: AGCTGTTTGTTGGCGGAAT |
| si- IGF2BP2-1 | Target sequences: GCGAAAGGAUGGUCAUCAUTT |
| si- IGF2BP2-2 | Target sequences: UGAAGCUGGAAGCGCAUAUTT |
| si- HNRNPR -1 | Target sequences: ATTAAACTCCCTGAGAGCATC |
| si- HNRNPR -2 | Target sequences: CAAGGTGCAAGAGTCCACAA |
| anti-miR-373-3p | Target sequences: ACACCCCAAAAUCGAAGCACUUC |

**Supplementary Table S3: Data of antibodies used in our research**

| Antibody | WB | IP | Specificity | Company |
| --- | --- | --- | --- | --- |
| Ly6G | - | 1:100 | Rabbit monoclonal | Cell Signaling Technology |
| Fibronectin (ab2413) | - | 1:100 | Rabbit polyclonal | Abcam |
| α-SMA (#19245) | - | 1:200 | Rabbit monoclonal | Cell Signaling Technology |
| HNRNPA2B1 (ab259894) | 1:1000 | - | Rabbit monoclonal | Abcam |
| Histone H3 (ab1791) | 1:1000 |  | Rabbit polyclonal | Abcam |
| GAPDH  (60004-1-lg) | 1:1000 |  | Mouse monoclonal | Proteintech |
| Alix (ab117600) | 1:500 |  | Mouse monoclonal | Abcam |
| TGFβRⅡ (ab259360) | 1:1000 | 1:30 | Rabbit monoclonal | Abcam |
| pSmad2/3 (ab254407) | 1:1000 | 1:50 | Rabbit monoclonal | Abcam |
| Smad2/3  (ab202445) | 1:1000 | 1:200 | Rabbit monoclonal | Abcam |

**Supplementary Table S4:** **Expression of MIR181A1HG in normal colorectal mucosa and primary cancerous tissues. (n=90)**

| Tissue sample | Expression of MIR181A1HG | | | | | *P* value | |
| --- | --- | --- | --- | --- | --- | --- | --- |
|  | | Low expression (n, %) | | High expression (n, %) | | |  |
| Normal mucosa | 64 (71.1) | | 26 (28.9) | | <0.001* | | |
| Tumor tissue | 41 (45.6) | | 49 (54.4) | |  | | |

* The significant difference in the expression of MIR181A1HG between normal colorectal mucosa and cancerous tissues.

**Supplementary Table S5: Associations between MIR181A1HG expression and clinicopathological characteristics in 90 CRC patients.**

| Variable | N | MIR181A1HG expression | | *P* value |
| --- | --- | --- | --- | --- |
|  | 90 | Low expression (41) | High expression (49) |  |
| Age (yr) |  |  |  | 0.576 |
| <57 | 44 | 20 (45.5%) | 24 (54.5%) |  |
| >=57 | 46 | 21 (45.7%) | 25 (54.3%) |  |
| Gender |  |  |  | 0.305 |
| Male | 52 | 22 (42.3%) | 30 (57.7%) |  |
| Female | 38 | 19 (50.0%) | 19 (50.0%) |  |
| Tumor location |  |  |  | 0.254 |
| Right | 49 | 27 (55.1%) | 22 (44.9%) |  |
| Transverse | 19 | 6 (31.6%) | 13 (68.4%) |  |
| Left | 3 | 2 (66.7%) | 1 (33.3%) |  |
| Sigmoid  Rectum | 3  16 | 1 (33.3%)  5 (31.3%) | 2 (66.7%)  11 (68.8%) |  |
| T classification |  |  |  | <0.001* |
| T 1 | 1 | 0 (0.0%) | 1 (100.0%) |  |
| T 2 | 18 | 17 (94.4%) | 1 (5.6%) |  |
| T 3 | 9 | 4 (44.4%) | 5 (55.6%) |  |
| T 4 | 62 | 20 (32.3%) | 42 (67.7%) |  |
| N classification |  |  |  | 0.003* |
| N 0 | 33 | 22 (66.7%) | 11 (33.3%) |  |
| N 1 | 28 | 12 (42.9%) | 16 (57.1%) |  |
| N 2 | 29 | 7 (24.1%) | 22 (75.9%) |  |
| M classification |  |  |  | <0.001* |
| M 0 | 51 | 37 (72.5%) | 14 (27.5%) |  |
| M 1 | 39 | 4 (10.3%) | 35 (89.7%) |  |
| AJCC stage |  |  |  | <0.001* |
| I | 16 | 15 (93.8%) | 1 (6.3%) |  |
| II | 12 | 6 (50.0%) | 6 (50.0%) |  |
| III | 23 | 16 (69.6%) | 7 (30.4%) |  |
| IV | 39 | 4 (10.3%) | 35 (89.7%) |  |
| Differentiation |  |  |  | 0.755 |
| Well | 3 | 1 (33.3%) | 2 (66.7%) |  |
| Moderate | 67 | 32 (47.8%) | 35 (52.2%) |  |
| Poor | 20 | 8 (40.0%) | 12 (60.0%) |  |

* Significant difference.

**Supplementary Table S6: Clinicopathological characteristics of 20 CRC patients with LM.**

| Variable | N |
| --- | --- |
|  | 20 |
| Age (yr) |  |
| <57 | 8 |
| >=57 | 12 |
| Gender |  |
| Male | 14 |
| Female | 6 |
| Tumor location |  |
| Right | 15 |
| Transverse | 2 |
| Left | 1 |
| Sigmoid  Rectum | 2  16 |
| T classification |  |
| T 1 | 0 |
| T 2 | 0 |
| T 3 | 6 |
| T 4 | 14 |
| N classification |  |
| N 0 | 0 |
| N 1 | 2 |
| N 2 | 18 |
| M classification |  |
| M 0 | 0 |
| M 1 | 15 |
| AJCC stage |  |
| I | 0 |
| II | 0 |
| III | 0 |
| IV | 15 |
| Differentiation |  |
| Well | 0 |
| Moderate | 7 |
| Poor | 13 |
